# On-line identification of the chemical constituents of Polygoni Multiflori Radix by UHPLC-Q-ToF MS/MS

**DOI:** 10.3389/fchem.2023.1158717

**Published:** 2023-04-21

**Authors:** Xueting Wang, Jianbo Yang, Xianlong Cheng, Ying Wang, Huiyu Gao, Yunfei Song, Feng Wei, Shuangcheng Ma

**Affiliations:** ^1^ Institute for Control of Chinese Traditional Medicine and Ethnic Medicine, National Institutes for Food and Drug Control, Beijing, China; ^2^ Xinjiang Uygur Autonomous Region Drug Inspection and Research Institute NMPA Key Laboratory for Quality Control of Traditional Chinese Medicine (Uyghur) Medicine Urumqi, Urumqi, China

**Keywords:** Polygoni Multiflori Radix, mass spectrometry, hepatotoxicity, identification, chemical components

## Abstract

**Introduction:** Polygoni Multiflori Radix (PMR) is a type of Chinese herbal medicine with rich chemical composition and pharmacological activity used widely in medicine and food. However, in recent years, there have been increasing numbers of negative reports about its hepatotoxicity. Identification of its chemical constituents for quality control and safe use is very important.

**Methods:** Three solvents of different polarities (water, 70% ethanol, and 95% ethanol solution) were used to extract the compounds from PMR. Extracts were analyzed and characterized by ultra-high-performance liquid chromatography-quadrupole time-of-flight mass spectrometry (UHPLC-Q-ToF MS/MS) in the negative-ion mode.

**Results:** 152 compounds were detected and identified: 50 anthraquinones, 33 stilbene derivatives, 21 flavonoids, seven naphthalene compounds, and 41 other compounds. Eight other compounds were reported for the first time in the PMR-related literature, and eight other compounds were potentially new compounds.

**Discussion:** This study lays a solid foundation for the screening of toxicity and quality-control indicators of PMR.

## 1 Introduction

In recent years, traditional Chinese medicine (TCM) has attracted increasing attention from researchers due to its diverse medicinal uses, mild efficacy, and few side effects. TCM-related products have been applied efficiently in medical treatment, food, cosmetics, and other fields.

Polygoni Multiflori Radix (He Shou Wu) (PMR) is derived from the dried root tubers of *Polygonum multiflorum* Thunb., which is a medicinal plant of the Polygonaceae family. It is used widely in medicines, food, and healthcare products. PMR can remove toxicity to eliminate carbuncles, prevent malaria, and moisten the bowel. It can also be used to treat pruritus caused by rubella, body deficiency due to malaria, and bowel dryness due to constipation (State Pharmacopoeia Commission. 2020).

PMR contains stilbene derivatives, anthraquinones, flavonoids, and polyphenols, which have significant effects against aging and tumors, protect nerves, and improve vascular function (Lin et al., 2015; Zhou et al., 2020; Ning et al., 2021; Teka et al., 2021; Wang et al., 2021). However, in recent years, adverse reactions after the clinical use of PMR (related mainly to hepatotoxicity using a normal dose) have focused attention on the safe use and quality control of PMR (Xia et al., 2017; Gong et al., 2019; Yang et al., 2021; Kong et al., 2022). The main compounds thought to cause the hepatotoxicity of PMR are anthraquinones, dianthraones, stilbene glycosides, and tannins (Yu et al., 2017; Kang et al., 2022; Yang et al., 2022).

PMR, as a type of TCM formulation, has multiple components, multiple signaling pathways, and multiple targets. Therefore, before carrying out an in-depth analysis of the toxic or active components of PMR, a comprehensive understanding of its chemical constituents is crucial. Previously, we showed that the order of hepatotoxicity of PMR extracts using different polar solvents in a zebrafish model was 70% ethanol extract >95% ethanol extract > water (Yang et al., 2018). Therefore, to comprehensively analyze and compare the components in different solvents, water, 70% ethanol, and 95% ethanol were used to extract the ingredients in PMR, and then an efficient method of ultra-high-performance liquid chromatography-quadrupole time-of-flight mass spectrometry (UHPLC-Q-ToF MS/MS) was established to analyze them.

## 2 Material and methods

### 2.1 Chemicals and reagents

Formic acid used for LC-MS (LC-MS grade) was purchased from Honeywell (Shanghai, China). Methanol (LC-MS grade) was obtained from MilliporeSigma (Burlington, MA, United States). Ultra-pure water was sourced from a water purification system (Alpha-Q; Millipore, Waltham, MA, United States). A microporous membrane (0.22 μm) was purchased from Dikma Technologies (Foothill Ranch, CA, United States). All the other reagents were analytically pure.

*N*,*N*-dimethyl-tryptophan methylester (**S1**), *N*-*trans*-feruloyltyramine (**S2**), *N*-*trans*-feruloyl-3-methyldopamine (**S3**), *cis*-emodin-physcion bianthrone (**S8**), multiflorumiside A (**S9**), polygonibene A (**S13**), 2,3,5,4′-tetrahydroxystilbene-2-O-(2″-feruloyl)-*β*-D-glucopyranoside (**S14**), polygonimitin E (**S18**), torachrysone-8-O-*β*-D-glucoside (**S19**), and *p*-hydroxy benzaldehyde (**S21**) were isolated by our research team. The purity of these compounds was >98%.

Emodin (**S7**; lot: 110758–201913), polydatin (**S11**; 111575–201603), *trans*-2,3,5,4′-tetrahydroxystilbene-2-O-*β*-D-glucoside (**S12**; 110844–201915), catechin (**S15**; 110878–201703), epicatechin (**S16**; 110877–202005), quercetin (**S17**; 100818–201610), and gallic acid (**S20**; 110831–201906) were purchased from China National Institute for Food and Drug Control (Beijing, China). Emodin-1-O-*β*-D-glucoside (**S4**; S28HB196292), emodin-8-O-*β*-glucoside (**S5**; ST10890126), physcion-8-O-*β*-D-glucoside (**S6**; ST23870105), and *cis*-2,3,5,4′-tetrahydroxystilbene-2-O-*β*-D-glucoside (**S10**; M31GB150098) were sourced from Shanghai Yuanye Biotechnology (Shanghai, China). The purity of these compounds was >95%.

### 2.2 Plant materials and apparatus

The dried roots of *Polygonum multiflorum* Thunb. were acquired from Deqing County in Zhaoqing City (Guangdong Province, China). We used a UPLC-QTOF-MS/MS system (1290 series) obtained from Agilent Technologies (Santa Clara, CA, United States). A 1/10,000 electronic analytical balance (XPE105) and a 1/1000 electronic analytical balance (Me 303/02) were obtained from Mettler Toledo (Geneva, Switzerland). An ultrasonic cleaner (Kq-300da) was obtained from Kunshan Ultrasonic Instruments (Beijing, China). A Lingsheng multi-functional grinder (C800) was obtained from Yongkang Hongsun Electromechanical (Yongkang, China). The MassHunter™ Workstation (version B.04.00) was obtained from Agilent Technologies.

### 2.3 Preparation of control samples and standard samples

Before extraction, dried crude medicinal materials were pulverized and screened through a 65-mesh sieve. Then, pulverized PMR (1 g) was weighed accurately in triplicate using an electronic analytical balance. Then, 20 mL of solvent (water, 70% ethanol, or 95% ethanol) was added. The mixture underwent ultrasound treatment for 20 min and was filtered with a polytetrafluoroethylene membrane (0.22 μm). PMR samples extracted with different solvents were obtained.

The 22 standard reference substances mentioned in Section 2.1 were dissolved in methanol separately and prepared into reference solutions of 0.5 mg/mL. According to the category of the compound, **S1–S3** reference solutions were mixed in equal proportions to prepare a mixed reference solution containing alkaloids. In the same way, **S4–S9** reference solutions, **S10–S15** reference solutions, and **S16–S22** reference solutions were mixed separately to prepare reference solutions containing anthraquinones, stilbene derivatives, and other compounds, respectively.

### 2.4 LC-MS conditions

LC was undertaken on a UHPLC system (1290; Agilent Technologies) equipped with a ZORBAX SB-C18 RRHD column (2.1 mm × 100 mm, 1.8 µm; Agilent Technologies) at 35°C. The mobile phase comprised methanol (A) and water containing 0.1% formic acid (B). The gradient program was as follows: 0–5 min, 25%–30% A; 5–15 min, 30%–40% A; 15–18 min, 40%–50% A; 18–23 min, 50%–55% A; 23–30 min, 55%–65% A; 30–35 min, 65%–85% A; 35–40 min, 85% A; 40–50 min, 85%–90% A; 50–60 min, 90% A; 60–61 min, 90%–100% A; 61–70 min, 100% A. The flow rate was 0.25 mL/min. The injection volume was 1 µL.

MS was undertaken on an Accurate-Mass Q-TOF/MS system (6520; Agilent Technologies) with an electrospray ionization source. Mass spectra were acquired in negative mode. The parameters were as follows: mass range = 100–1500 m*/z*; scanning rate = 3 spectra/s; capillary voltage = 3.5 kV; nozzle voltage = 1 kV; fragmentor voltage = 150 V; cone voltage = 65 V; drying temperature = 350°C; flow rate = 0.3 mL/min. Data were obtained by data-dependent MS/MS (i.e., in each cycle, the top two precursor ions were chosen for fragmentation at a collision energy of 0, 10, and 20 V). Data were recorded and processed using the MassHunter Workstation.

### 2.5 Data analyses

The MassHunter software was used to extract the base peak chromatogram (BPC) of a compound. MS data provided the precise molecular weight, molecular formula, isotope abundance ratio, and error range for chromatography. MS/MS data provided abundant information on fragment ions.

To evaluate the chemical components in PMR systematically and rapidly, some analytical methods were used. First, the cracking pathways of reference substances were analyzed and summarized by MS, which is useful for structural speculations of compounds of the same type. Second, our in-house MS database and MS/MS databases that include the chemical names, molecular formulae, accurate molecular mass, chemical structures, and fragment ions were established by searching the related literature and compound databases (ScienceDirect (Elsevier), ChemSpider, PubMed, Chinese National Knowledge Infrastructure, SciFinder, and PubChem). Based on the reference substance and our in-house database, compounds were identified with mass errors of <5 ppm for the precursor and one or more products. Feature extraction was used to identify compounds with a weak response signal and confluence.

**FIGURE 1:**
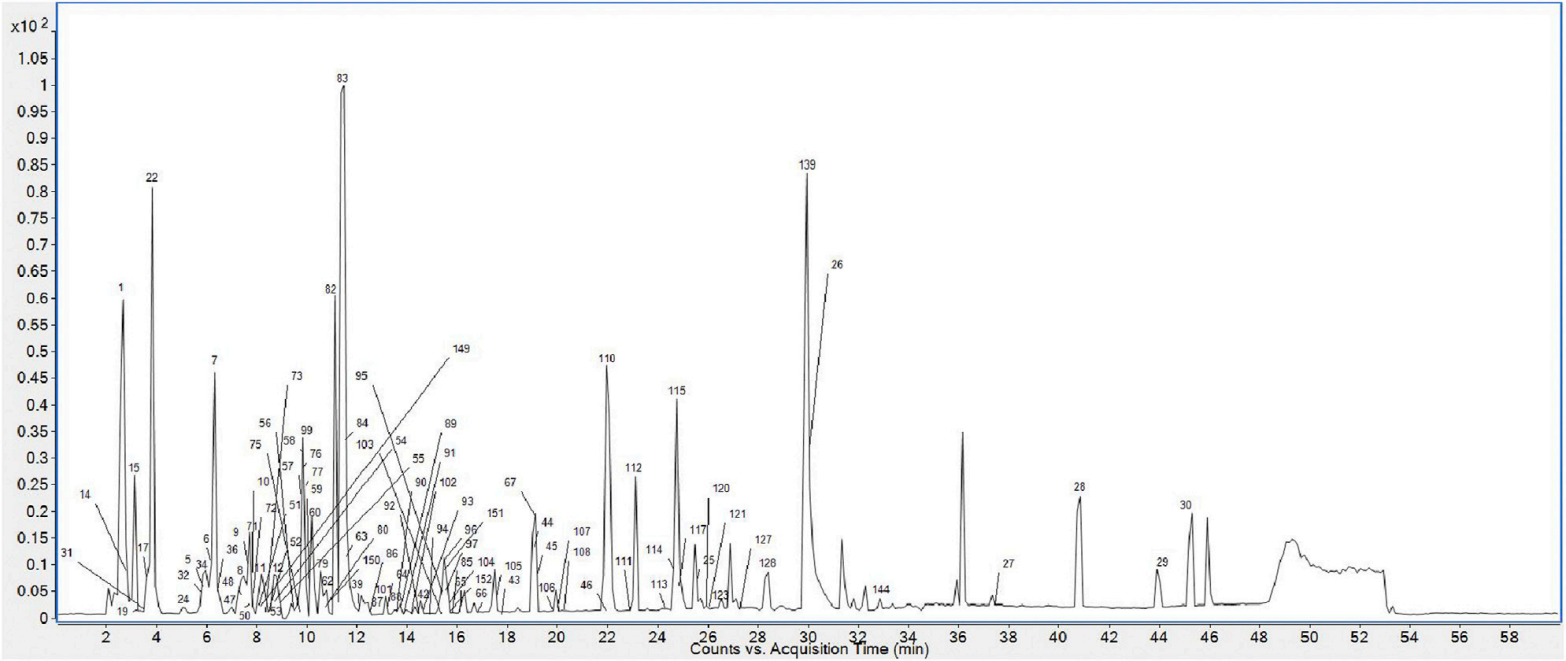
BPC of PMR 70% ethanol extract.

**FIGURE 2:**
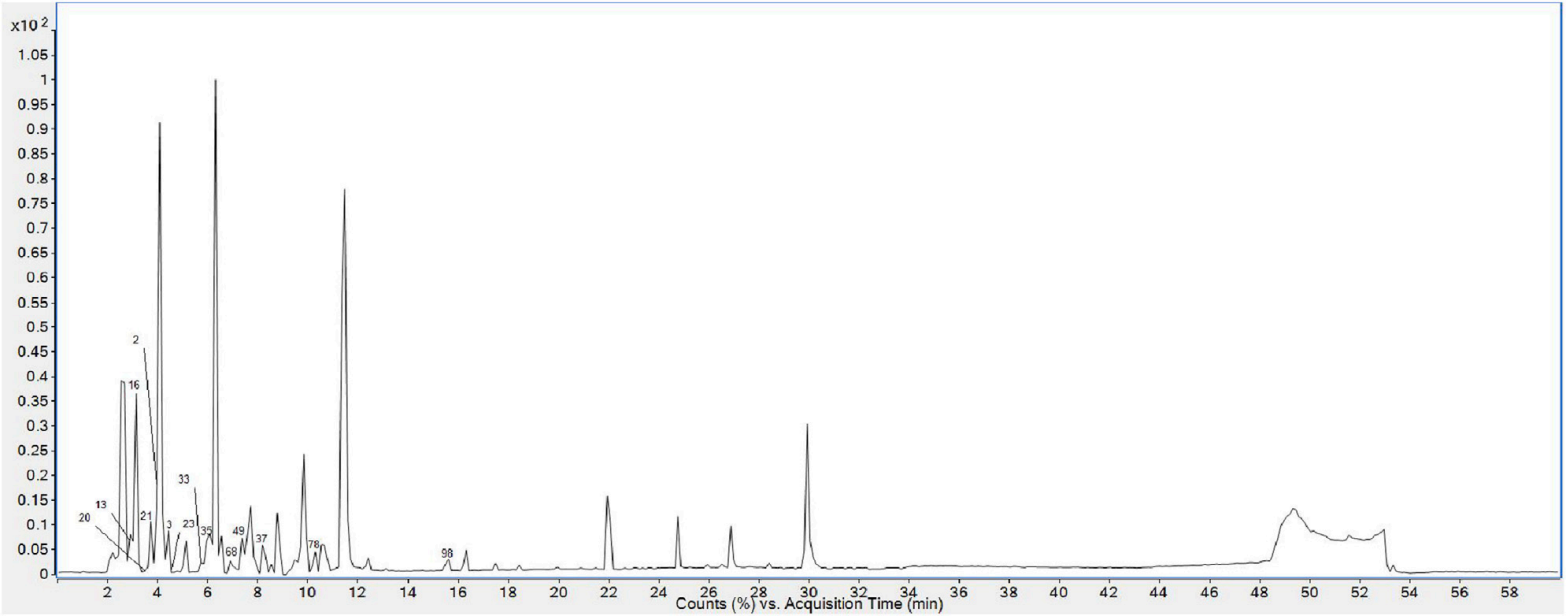
BPC of PMR water extract.

**FIGURE 3:**
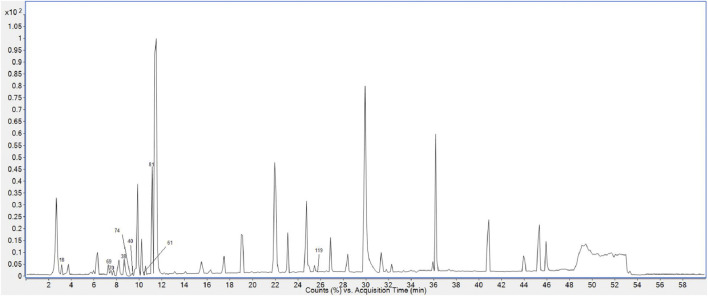
BPC of PMR 95% ethanol extract.

Finally, 158 compounds were identified: 50 anthraquinones, 31 stilbene derivatives, 21 flavonoids, seven naphthalene compounds, and 49 other compounds. The BPCs of different sample solutions are shown in Figures 1–3. Feature-extraction chromatograms and BPCs of mixed reference solutions are revealed in Supplementary Materials. The specific information of reference substances and identified compounds is given in Table 1 and Supplementary Table S1.

**TABLE 1:** Mass data of 21 reference substances.

Peak	t_R_ (min)	Identification	Proposed formula [M-H]-	Measured mass [M-H]^-^	Accuracy mass [M-H]^-^	Error (ppm)	MS/MS	Classification
S1	8.32	N,N-dimethyl-tryptophan methylester	C_14_H_17_N_2_O_2_^−^	245.1297	245.1296	0.4	142.0663[M-H-C_2_H_3_O_2-_C_2_H_6_N]^-^; 186.0560[M-H-C_2_H_3_O_2_]^-^	Alkaloids
S2	15.32	N-trans-feruloyltyramine	C_18_H_18_NO_4_^−^	312.1240	312.1242	0.6	297.1007[M-H-CH_3_]^-^; 178.0510[M-H-C_8_H_6_O_2_]^-^; 148.0530[M-H-C_9_H_10_NO_2_]^-^	Alkaloids
S3	15.77	N-trans-feruloyl-3-methyldopamine	C_19_H_20_NO_5_^−^	342.1345	342.1347	0.6	327.1112[M-H-CH_3_]^-^; 178.0508[M-H-CH_3_-C_10_H_13_O_2_]^-^; 148.0528[M-H-C_10_H_12_NO_3_]^-^	Alkaloids
S4	17.56	Emodin-1-O-*β*-glucoside	C_21_H_19_O_10_^−^	431.0984	431.0984	0	269.0455[M-H-C_6_H_10_O_5_]^-^; 240.0425[M-H-C_6_H_10_O_5_-CHO]^-^	Anthraquinones
S5	21.95	Emodin-8-O-*β*-glucoside	C_21_H_19_O_10_^−^	431.0986	431.0984	0.4	413.0871[M-H-H_2_O]^-^; 311.0560[M-H-C_4_H_8_O_4_]^-^; 269.0456[M-H-C_6_H_10_O_5_]^-^;	Anthraquinones
S6	24.78	Physcion-8-O-*β*-glucoside	C_22_H_21_O_10_^−^	445.1128	445.1140	2.7	380.9766 431.0974 283.0613[M-H-C_6_H_10_O_5_]^-^	Anthraquinones
S7	29.89	Emodin	C_15_H_9_O_5_^−^	269.0454	269.0456	0.7	225.0557[M-H-CO-CO]^-^;	Anthraquinones
S8	34.14	Cis-emodin-physcion bianthrone	C_31_H_23_O_8_^−^	523.1397	523.1398	0.2	254.0585[M-H-C_16_H_13_O_4_]^-^	Anthraquinones
S9	7.79	Multiflorumiside A	C_40_H_43_O_18_^−^	811.2453	811.2455	0.3	649.1831[M-H-C_6_H_10_O_5_]^-^; 487.1307[M-H-C_6_H_10_O_5_-C_6_H_10_O_5_]^-^; 405.1198[M-H-C_20_H_22_O_9_]^-^; 243.0664[M-H-C_20_H_22_O_9_-C_6_H_10_O_5_]^-^	Stilbene derivatives
S10	9.95	Cis-2,3,5,4′-tetrahydroxystilbene-2-O-D-glucoside	C_20_H_21_O_9_^−^	405.1192	405.1191	0.2	243.0664[M-H-C_6_H_10_O_5_]^-^	Stilbene derivatives
S11	10.88	Polydatin	C_30_H_29_O_12_^−^	389.1243	389.1242	0.3	227.0717[M-H-C_6_H_10_O_5_]^-^	Stilbene derivatives
S12	11.38	Trans-2,3,5,4′-tetrahydroxystilbene-2-O-D-glucoside	C_20_H_21_O_9_^−^	405.1191	405.1192	0.2	243.0664[M-H-C_6_H_10_O_5_]^-^	Stilbene derivatives
S13	12.72	Polygonibene A	C_40_H_41_O_18_^−^	809.2299	809.2298	0.1	647.1767[M-H-C_6_H_10_O_5_]^-^; 485.1237[M-H-C_6_H_10_O_5_-C_6_H_10_O_5_]^-^; 405.1246[M-H-C_20_H_20_O_9_]^-^; 243.0658[M-H-C_20_H_20_O_9_-C_6_H_10_O_5_]^-^	Stilbene derivatives
S14	15.31	2,3,5,4′-tetrahydroxystilbene-2-O-(2″-feruloyl)-*β*-D-glucopyranoside	C_30_H_29_O_12_^−^	581.1667	581.1664	0.5	405.1187[M-H-C_10_H_8_O_3_]^-^; 387.1090[M-H-C_10_H_8_O_3_-H_2_O]^-^; 337.0918[M-H-C_14_H_12_O_4_]^-^; 243.0661[M-H-C_10_H_8_O_3_-C_6_H_10_O_5_]^-^; 175.0404[M-H-C_10_H_22_O_9_]^-^	Stilbene derivatives
S15	8.27	Catechin	C_15_H_13_O_6_^−^	289.0720	289.0718	0.7	245.0820[M-H-CO_2_]^-^; 203.0715[M-H-CO_2_-C_2_H_2_O]^-^; 179.0350[M-H-C_6_H_6_O_2_]^-^; 151.0401[M-H-C_6_H_6_O_2_-CO]^-^; 125.0245[M-H-C_9_H_8_O_3_]^-^; 109.0295[M-H-C_9_H_8_O_3_-O]^-^;	Flavonoids
S16	9.44	Epicatechin	C_15_H_13_O_6_^−^	289.0720	289.0718	0.7	245.0820[M-H-CO_2_]^-^; 203.0715[M-H-CO_2_-C_2_H_2_O]^-^; 179.0350[M-H-C_6_H_6_O_2_]^-^; 151.0401[M-H-C_6_H_6_O_2_-CO]^-^; 125.0245[M-H-C_9_H_8_O_3_]^-^; 109.0295[M-H-C_9_H_8_O_3_-O]^-^;	Flavonoids
S17	18.22	Quercetin	C_15_H_9_O_7_^−^	301.0357	301.0354	1.0	273.0404[M-H-CO]^-^; 257.0455[M-H-CO_2_]^-^; 151.0030[M-H-C_8_H_6_O_3_]^-^;	Flavonoids
S18	17.65	Polygonimitin E	C_25_H_31_O_13_^−^	539.1770	539.1770	0	245.0818[M-H-C_6_H_10_O_5_]^-^; 230.0583[M-H-C_6_H_10_O_5_-CH_3_]^-^	Naphthalenes
S19	19.14	Torachrysone-8-O-*β*-D-glucoside	C_20_H_23_O_9_^−^	407.1346	407.1348	0.5	245.0818[M-H-C_11_H_18_O_9_]^-^; 230.0679[M-H-C_11_H_18_O_9_-CH_3_]^-^	Naphthalenes
S20	6.27	Gallic acid	C_7_H_5_O_5_^−^	169.0140	169.0142	1.2	151.0033[M-H-H_2_O]^-^; 125.0245[M-H-CO_2_]^-^; 107.0137[M-H-CO_2_-H_2_O]^-^	Others
S21	10.56	p-Hydroxy benzaldehyde	C_7_H_5_O_2_^−^	121.0294	121.0295	0.8		Others

## 3 Results

### 3.1 Analysis of flavonoids

In a broad sense, flavonoids are formed by two benzene rings connected to each other by three carbon atoms (C6–C3–C6). Flavonoids have antioxidant and free radical-scavenging activity (Aziz et al., 2022; Shen et al., 2022). Depending on the substitution patterns and position of two benzene rings and the oxidation state of the heterocyclic-C3 ring, flavonoids can be classified into seven major subclasses: flavan-3-ols, flavones, flavonols, flavanones, anthocyanins, chalcones, and isoflavonoids (Dretcanu et al., 2022). Here, we also discuss proanthocyanidins as oligomers of (epi) catechin units.

We obtained three reference substances of flavonoids, namely, catechin, epicatechin, and quercetin. Catechin and epicatechin were optical isomers with the structural framework of flavanols. Catechin and epicatechin were eluted at 8.266 min and 9.441 min, respectively. They possessed the same precursor ions at *m/z* 289.0720 (C_15_H_13_O_6_) and the same main characteristic fragment ions at *m/z* 151 (C_8_H_7_O_3_) and *m/z* 125 (C_6_H_5_O_3_), which were produced by RDA (retro Diels–Alder reaction) rearrangement and heterocyclic cracking, along with 138 Da (C_7_H_6_O_3_) and 164 Da (C_9_H_8_O_3_) neutral loss (March and Brodbelt, 2008). So, fragment ions at *m/z* 289.0720 (C_15_H_13_O_6_), *m/z* 151 (C_8_H_7_O_3_), and *m/z* 125 (C_6_H_5_O_3_) could be taken as diagnostic ions of flavanols. Differently, the quercetin reference substance had a flavonol skeleton, which has an additional carbon–carbon double bond than the flavanones. It was eluted at 18.216 min, and its [M-H]^−^ was at *m/z* 301.0357 (C_15_H_9_O_7_). In addition to forming the same daughter ion at *m/z* 151 (C_8_H_7_O_3_) as (epi) catechin, quercetin reference substance also produced other characteristic fragment ions at *m/z* 273 (C_14_H_9_O_6_) and *m/z* 257 (C_14_H_9_O_5_) by losing CO (28 Da) and CO_2_ (44 Da). The typical cracking pathway of flavonoids also involved the loss of CH_3_ (15Da) or H_2_O (18 Da). For other flavonoids, such as flavonoid glycosides or galloyl-flavonoids, fragmentation was characterized by the loss of glucose or galloyl along with the production of many skeleton fragment ions (March and Brodbelt, 2008; Vukics and Guttman, 2010; Aziz et al., 2022).

**FIGURE 4:**
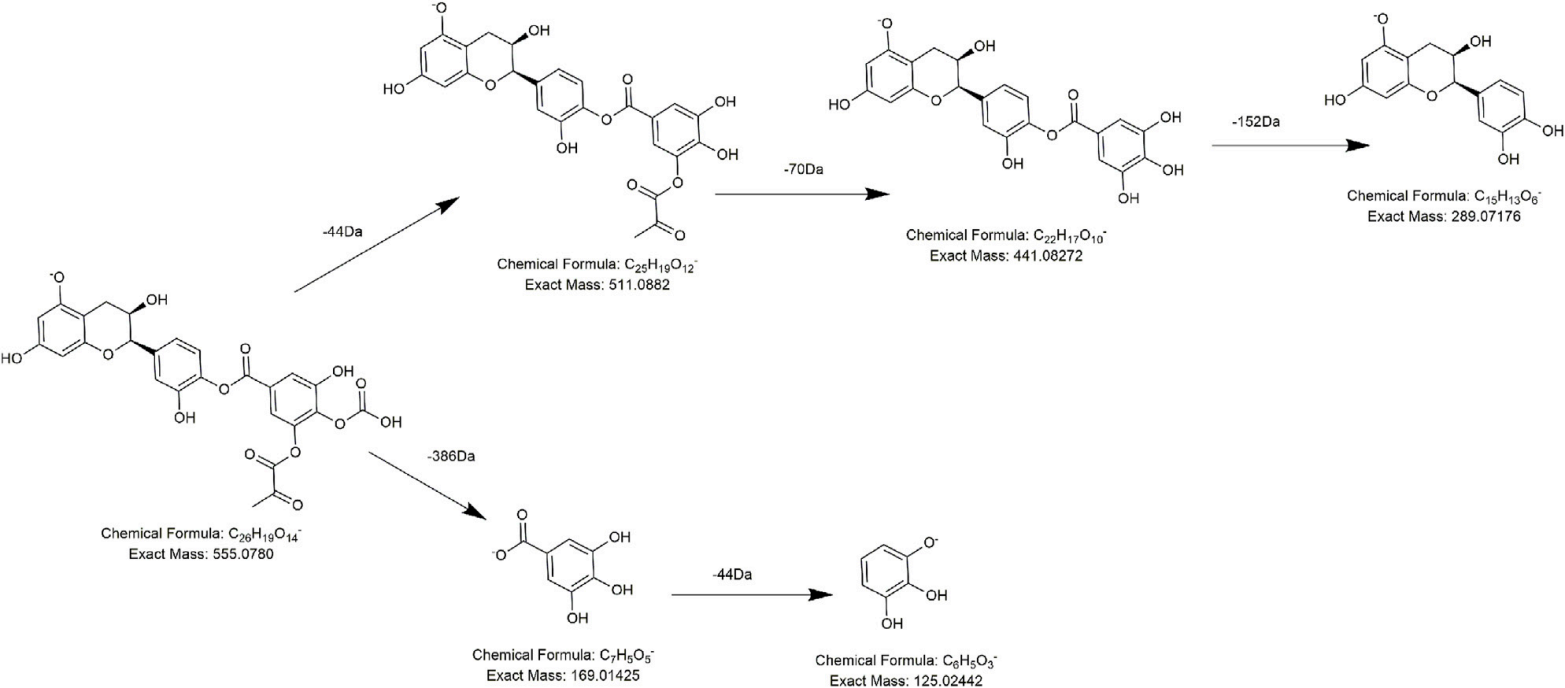
Proposed fragmentation pathways of **FL15** (new compound-3).

According to the retention time and MS/MS information, compounds **FL6** and **FL10** were identical to the catechin standard substance and epicatechin standard substance, respectively. Compounds **FL14**, **FL15,** and **FL16** had precursor ions at *m/z* 441.0829 (C_22_H_17_O_10_), *m/z* 555.0756 (C_24_H_19_O_14_), and *m/z* 693.1825 (C_35_H_33_O_15_), respectively, and were presumed to be catechin derivatives because they all produced fragment ions at *m/z* 289 (C_15_H_13_O_6_) and *m/z* 125 (C_6_H_5_O_3_). Taking compound **FL14** (which was eluted at 10.3 min) as an example, with a precursor ion at *m/z* 441.0829, it not only lost 152 Da (C_8_H_8_O_3_ ) to produce a fragment ion at *m/z* 289 (C_15_H_13_O_6_) but also possessed a daughter ion at *m/z* 169 (C_7_H_5_O_5_), which is a typical fragment of a galloyl substituent. Therefore, we presumed that **FL14** was formed from the combination of (epi) catechin and galloyl substituents, that is, catechin galloyl ester (Wang et al., 2021). Similarly, except for the typical diagnostic ions of flavonoids (i.e., at *m/z* 289, m*/z* 125, and *m/z* 169), compound **FL15** also generated the fragment ion at *m/z* 511.0865 (C_23_H_19_O_12_) by losing 44 Da (CO_2_), which suggested a carboxyl substituent. Also, in its subsequent collision, a neutral loss of 70 Da emerged to generate the fragment ion at *m/z* 441.0834 (C_20_H_17_O_10_), which implied a malonyl (C_3_H_2_O_2_) group. We preliminarily speculated that **FL15** consisted of an (epi) catechin nucleus and a molecule of carboxyl and a molecule of malonyl substitution. Tentative speculation of its structure and the lytic pathway is stated in Figure 4, which was conjectured to be a potential new compound. Compound **FL16** exhibited [M-H]^−^ at *m/z* 693.1824 (C_35_H_33_O_15_) with a retention time of 10.81 min, and its primary fragment ions were at *m/z* 289 (C_15_H_13_O_6_) and *m/z* 405 (C_29_H_23_O_10_). Hence, it was identified as a polygonflavanol A (C_35_H_34_O_15_): a combination of a tetrahydroxystilbene glycoside (C_29_H_24_O_10_) and (epi) catechin (C_15_H_14_O_6_) formed by opening the ene bond and forming a ring. (Huang et al., 2018). As for **FL1**, its excimer ion (*m/z* 305) was 16 Da higher than that of (epi) catechin, and one of the typical fragment ions *m/z* 125 was also generated, so it is reasonable to deem that it has one more hydroxyl group than (epi) catechin. According to the literature, compound **FL1** was speculated to be gallocatechin A (Wang et al., 2017).

In a similar manner, compound **FL18** was assumed to be an isomer of quercetin upon comparison with the eluting time and MS/MS information of quercetin’s standard substance. Meanwhile, several compounds share similar nuclear factors with quercetin. Take compound **FL11** as an example, after losing a molecule of hexose substitution (162 Da, C_6_H_10_O_5_), a unit of H_2_O (18 Da) was lost immediately, and **FL11** produced the fragment ions at *m/z* 287 and *m/z* 269. The two fragment ions were 14 Da and 32 Da lower than the typical diagnostic ions *m/z* 301 of quercetin, respectively. So, we believe that **FL11** has one less hydroxide radical (16 Da) and one unsaturation (−2 Da) more than quercetin, so it most likely processes flavonol structures with one less phenolic hydroxide radical than quercetin. Because of the aforementioned reasons, **FL11** was identified as dihydrokaempferol-3-glucoside (Huang et al., 2018).

**FIGURE 5:**
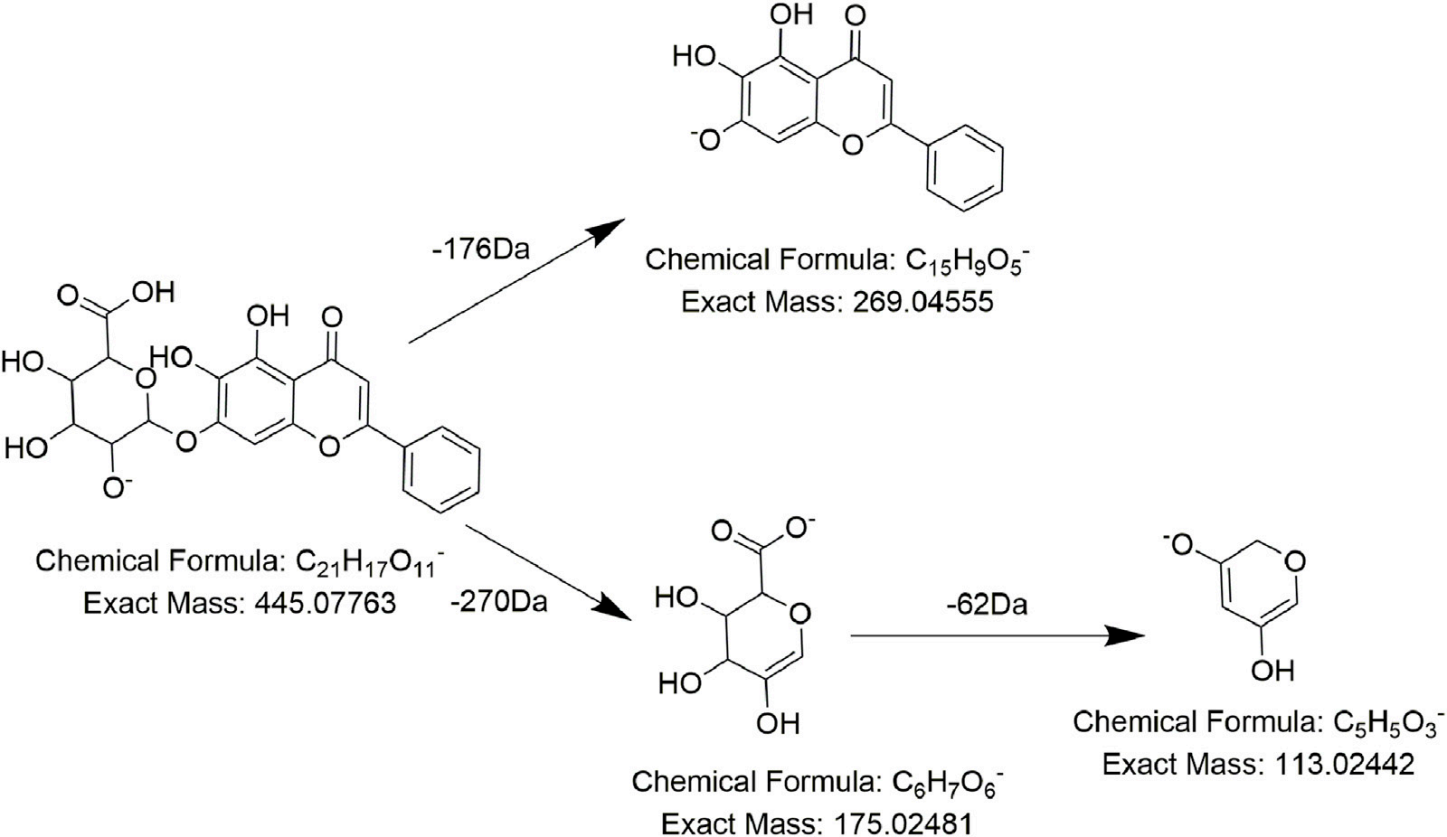
Proposed fragmentation pathways of **FL20** (baicalin).

Analogously, compound **FL20** displayed a precursor ion at *m/z* 444.0766 and presented typical fragment ions at *m/z* 269 after a 177 Da (C_6_H_8_O_6_) neutral loss. Combining with the daughter ions at *m/z* 113 and *m/z* 175, we believed that there is a glucuronic-group substitution (C_6_H_8_O_6_). So, *m/z* 269 was reasonably inferred to be the nucleus of compound **FL20**, which was the flavone nucleus. According to the literature (Wu et al., 2005), it was speculated to be baicalin (Figure 5), which was reported for the first time in PMR-related literature. Compound **FL19** was eluted at 16.1 min with the precursor ion at *m/z* 447.40493, and a comparison with compound **FL20** revealed its molecular weight to be 2 Da lower. The same as compound **FL20**, after losing 177 Da (C_6_H_8_O_6_), it generated the fragment ion at *m/z* 271 (269 Da +2 Da) and had fragment ions at *m/z* 175 and *m/z* 113. According to the literature, it was considered to be naringenin-7-O-glucuronide (Rui et al., 2020).

**FIGURE 6:**
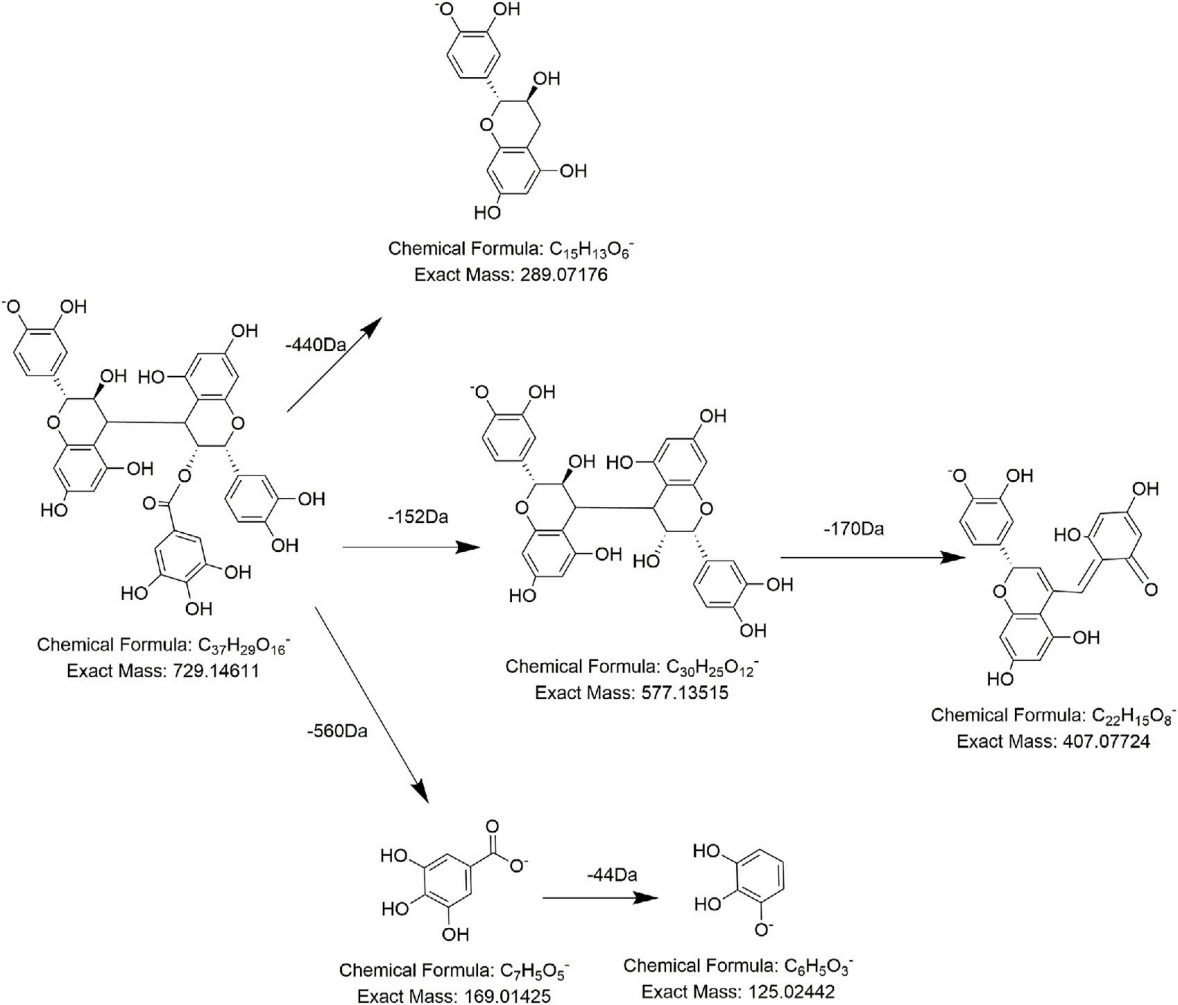
Proposed fragmentation pathways of **FL5** (3-O-galloyl-procyanidin).

As for procyanidins, which are made up of two units of flavanol, they tend to break the C–C bond, losing one unit of nuclear flavanols. During this procedure, 288 Da (C_15_H_12_O_6_) neutral loss and characteristic ions at m/z 289 (C_15_H_13_O_6_) are generated. Compounds **FL2**, **FL3**, **FL5**, **FL7**, and **FL8** not only presented the characteristic daughter ion at *m/z* 125 but also exhibited a typical neutral loss of 288 Da (C_15_H_12_O_6_). So, they were tentatively regarded as procyanidin derivatives. For example, compound **FL5** was detected at 8.11 min, with the precursor ion at *m/z* 729.1454 (C_37_H_29_O_16_). Under the collision energy in MS/MS, compound **FL5** lost 152 Da (C_8_H_8_O_3_) and 288 Da (C_15_H_12_O_6_) to produce fragment ions at *m/z* 577 (C_30_H_25_O_12_) and *m/z* 289 (C_15_H_13_O_6_) successively, which could be regarded as a decrease of one unit of galloyl and (epi) catechin structure. Therefore, according to the literature, compound **FL5** was speculated to be 3-O-galloyl-procyanidin B (Figure 6) (Qiu et al., 2013). Analogously, compound **FL2** and compound **FL9** displayed [M-H]^−^ at *m/z* 577.1349 (C_30_H_25_O_12_) and *m/z* 881.1571 (C_44_H_33_O_20_), of which one was 152-Da (C_8_H_8_O_3_) lower and one was 152-Da (C_8_H_8_O_3_) higher than the precursor ion of compound **FL5**. In addition, these three compounds shared the same daughter ions at *m/z* 729 (C_37_H_29_O_16_), *m/z* 577 (C_30_H_25_O_12_), and *m/z* 169 (C_7_H_5_O_5_). Hence, it was reasonable to assume that the three compounds differed by only one unit of a galloyl substituent (152 Da, C_8_H_8_O_3_) and that compound **FL2** and compound **FL9** might be procyanidin B and di-galloyl-procyanidin B, respectively (Qiu et al., 2013; Zhao et al., 2021). Compound **FL3** was predicted to be the catechin trimer procyanidin C1 by the same method (Zhao et al., 2021). The MS behavior of **FL7** and **FL8** was identical to that of **FL3** and **FL5,** except for the retention time, so we presumed them to be isomers of 3-O-galloyl-procyanidin B and procyanidin C1, respectively(Zhao et al., 2021).

**FIGURE 7:**
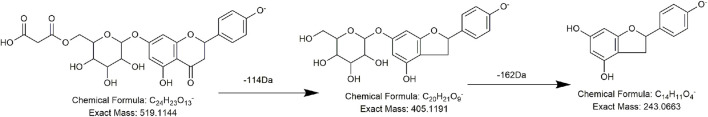
Proposed fragmentation pathways of compound **FL4** (new compound-2).

**FIGURE 8:**
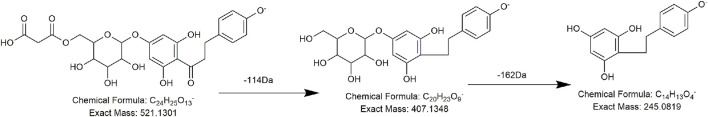
Proposed fragmentation pathways of compound **FL21** (new compound-4).

The precursor ions of compounds **FL4** and **FL21** were at *m/z* 519.1121 (C_24_H_23_O_13_) and *m/z* 521.1300 (C_24_H_25_O_13_), respectively, and in both of their MS/MS spectra, 114 Da (C_4_H_2_O_4_) and 162 Da (C_6_H_10_O_5_) were lost successively. So, we presumed that **FL4** and **FL21** process the same substitution, and during the MS/MS procedure, one unit of the malonyl group (86 Da, C_3_H_2_O_3_) plus one unit of CO (28 Da) and one unit of hexose (162 Da, C_6_H_10_O_5_) were lost in sequence. The difference in molecular weight (2 Da) might be due to the unsaturation of the parent nucleus. The proposed structure and pathway of compounds **FL4** and **FL21** are shown in Figures 7, 8, respectively. Compounds **FL12**, **FL13,** and **FL17** had the same molecular weight and fragment ions as compound **FL4** but not an identical retention time, so they were presumed to be conformational isomers of compound **FL4**. Compounds **FL4**, **FL12**, **FL13**, **FL17**, and **FL21** were reported for the first time in the PMR-related literature.

### 3.2 Analysis of stilbene derivatives

Stilbene derivatives are typical components in PMR. They have antibacterial, anti-inflammatory, anticancer, antioxidant, neuroprotective, and immunomodulatory effects (Lin et al., 2015; De Filippis et al., 2017; Lee et al., 2019). Stilbene derivatives in PMR that have been isolated so far include tetrahydroxystilbene and its glycoside, substituted tetrahydroxystilbene glycoside, a dimer of tetrahydroxystilbene and its glycoside, and a small amount of trihydroxy stilbene glycoside derivatives (Zhang and Cui, 2016; Yuan et al., 2017; Li et al., 2018; Nguyen et al., 2020).

Reference substances *cis*-2,3,5,4′-tetrahydroxystilbene-2-O-*β*-D-glucoside (*cis*-THSG) and *trans*-2,3,5,4′-tetrahydroxystilbene-2-O-*β*-D-glucoside (*trans*-THSG) were isomers with a retention time of 9.98 min and 11.5 min, respectively. Except for the retention time, their MS behavior was essentially identical with the precursor ion at *m/z* 405.1191 (C_20_H_21_O_9_) and the primary fragment ion at *m/z* 243 (C_14_H_11_O_4_), which was obtained by losing one unit of hexose (162 Da, C_14_H_11_O_4_) (Wang et al., 2021). Among substituted stilbene derivatives, the reference substance 2,3,5,4′-tetrahydroxystilbene-2-O-(2″-feruloyl)-*β*-D-glucopyranoside aided further identification of homologous series. Under the action of collision energy, 2,3,5,4′-tetrahydroxystilbene-2-O-(2″-feruloyl)-*β*-D-glucopyranoside yielded fragments at *m/z* 405.1191 (C_20_H_21_O_9_) and *m/z* 243 (C_14_H_11_O_4_), which corresponded with a tetrahydroxystilbene-glucoside nucleus. Other debris, including those at *m/z* 193.0506 (C_10_H_9_O_4_) and *m/z* 175.0401 (C_10_H_7_O_3_), corresponded to the feruloyl group. Reference substances multiflorumiside A and polygonibene A showed two different patterns of tetrahydroxystilbene dimers; they are both polymerized by forming a ring, but the ring atoms are in different positions. Multiflorumiside A was a dimer of THSG with a retention time of 7.86 min and a precursor ion at *m/z* 811.2455 (C_40_H_43_O_18_). After appropriate interaction with collision energy, the polymerization C–C bond was broken, a unit of THSG was removed (406 Da, C_20_H_22_O_9_), and a daughter ion at *m/z* 405 (C_20_H_21_O_9_) was generated, followed by the loss of one unit of hexose to produce another characteristic fragment ion at *m/z* 243 (C_14_H_11_O_4_). Reference substance polygonibene A was also a dimer of THSG but formed by the C–O bond and C–C bond. It was detected at 12.49 min and possessed the parent ion at *m/z* 809.2298 (C_40_H_41_O_18_). Apart from having the same cleavage pathway as multiflorumiside A, the glycosidic bond of polygonibene A was easier to break and produced fragment ions at *m/z* 647 (C_34_H_31_O_13_) and *m/z* 485 (C_28_H_21_O_8_), along with the shedding of two units of hexose (162 Da, C_14_H_11_O_4_). Different from all of the previously mentioned references, the reference substance polydatin was a trihydroxy stilbene glycoside and was composed of one unit of resveratrol and one unit of glucose. Hence, the representative rupture was identical to that of tetrahydroxystilbene-glucoside: loss of a glycosidic bond (162 Da, C_6_H_10_O_5_) to produce a fragment ion at *m/z* 227.0713 (C_14_H_11_O_3_).

Compared with the previously stated standard substance, compounds **D12** and **D18** were identified as *cis*-2,3,5,4′-tetrahydroxystilbene-2-O-*β*-D-glucoside and *trans*-2,3,5,4′-tetrahydroxystilbene-2-O-*β*-D-glucoside, respectively, by comparing molecular weight, retention time, and MS information. Similarly, compounds **D32** and **D33** were presumed to be tetrahydroxystilbene-O-(feruloyl)-hexose and its isomer. Compounds **D14**, **D22**, **D25,** and **D27** were identified as being isomers of polygonibene A. Compounds **D9**, **D11,** and **D19** were assumed to be isomers of multiflorumiside A. Compounds **D16** and **D26** were identified as polydatin and resveratrol, respectively.

**FIGURE 9:**
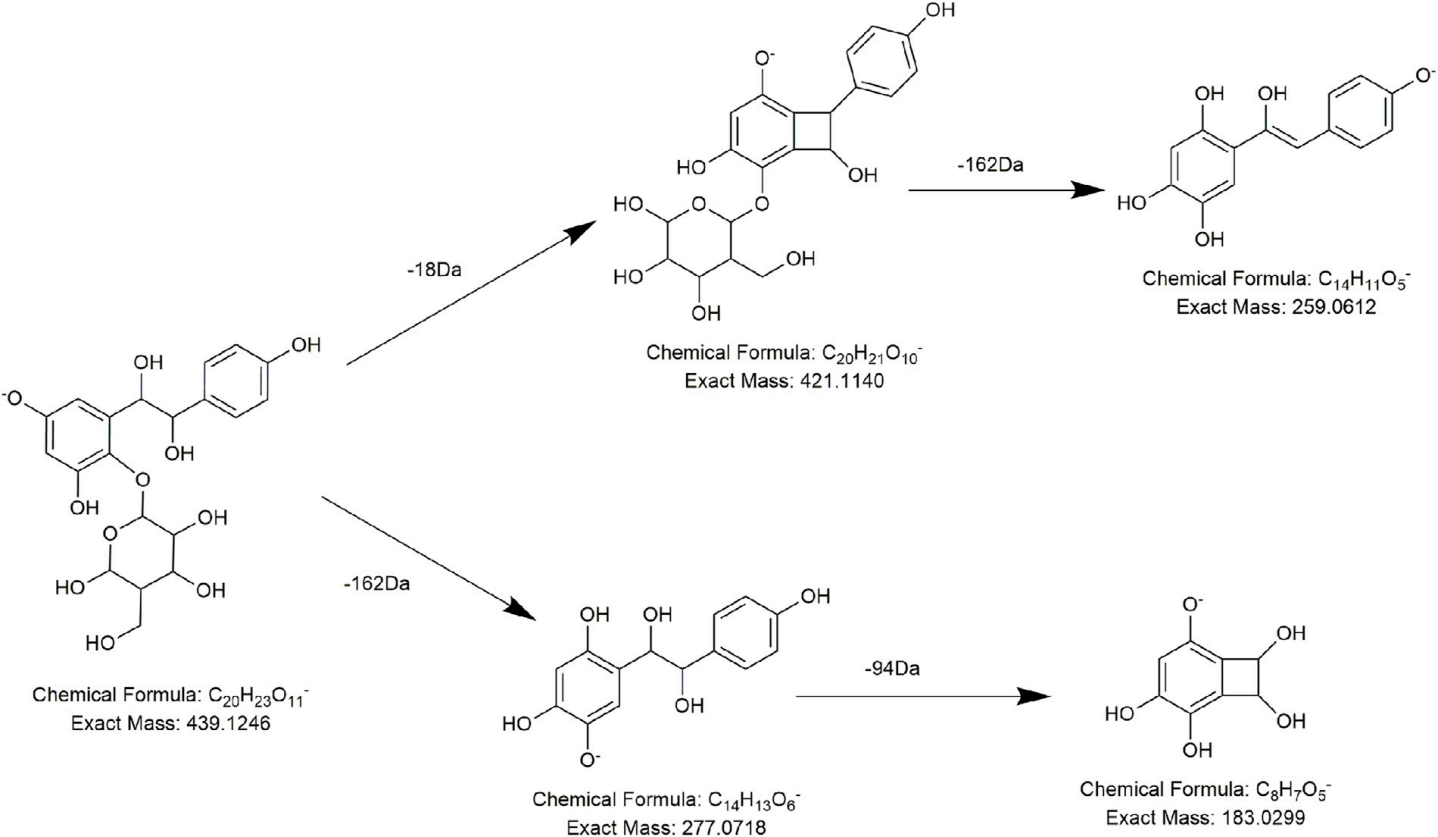
Proposed fragmentation pathways of **D1** (new compound-1).

According to the MS fragments of compound **D1**, we found its major fragment ion at *m/z* 259.0614, which was 16 Da higher than the characteristic fragment ion of stilbene derivatives (243 Da, C_14_H_11_O_4_). Combined with the consecutive neutral loss of 18 Da (H_2_O) and 162 Da (C_6_H_10_O_5_) from *m/z* 439.1246 to *m/z* 259.0614, we speculated that compound **D1** might be an oxidized derivative of tetrahydroxystilbene glucoside, which lost one molecule of water and one molecule of hexose in succession in the collision pool. Moreover, there was another lytic pathway, in which a unit of hexose and a unit of phenol were consecutively lost. In addition, specific elucidation of the two proposed cleavage methods is shown in Figure 9.

Similar to polygonibene A, compound **D3**, with a retention time of 6.87 min and precursor ion at *m/z* 827.2396 (C_40_H_43_O_19_), also generated the characteristic ion at *m/z* 485 (C_28_H_21_O_8_) under the effect of collision energy. Its molecular weight of 486 Da (C_28_H_22_O_8_) can be viewed as the addition of two molecules of tetrahydroxystilbene (243 Da, C_14_H_11_O_4_), and MS fragment information displayed that it lost two molecules of glucose in succession to generate *m/z* 485. Hence, in accordance with the literature, compound **D3** was preliminarily identified as the dimer of THSG formed in different ways: polygonumside C/D, which polymerizes by one unit of the C–C bond (Wang et al., 2017). Compounds **D4**, **D5**, **D6**, and **D8**, which had the same precursor ions and fragment ions but were eluted at different times, were referred to as isomers of polygonumside C/D.

Compounds **D10**, **D17**, **D20**, **D21**, **D23**, **D24**, **D28**, **D29**, **D30**, **D31**, **D32**, and **D33** produced the same typical fragment ions of THSG at *m/z* 405 (C_20_H_21_O_9_) by different types of neutral loss in MS/MS. Hence, it was rational to speculate that they were THSG derivatives formed by combining THSG with different substituents. For example, compounds **D10**, **D17**, and **D20** shared the same [M-H]^−^ at *m/z* 557.1299 (C_27_H_25_O_13_), and all lost 152 Da (C_7_H_4_O_4_) to produce the characteristic ion at *m/z* 405 (C_20_H_21_O_9_), so they were assumed to be THSG replaced by a galloyl group (152 Da, C_7_H_4_O_4_) (Xu et al., 2006; Qiu et al., 2013). For the same reason, compounds **D21** and **D23** with [M-H]^−^ at *m/z* 447.1297 (C_22_H_3_O_10_) were presumed to be THSG substituted by an acetyl group (42 Da, C_2_H_2_O) as its major neutral loss of 42 Da coincided perfectly with the molecular weight of the acetyl group (42 Da, C_2_H_2_O) (Liang et al., 2018); Compound **D24** and compound **D29**, with precursor ions at *m/z* 567.1506 (C_26_H_31_O_14_) and *m/z* 491.1191 (C_23_H_23_O_12_), respectively, were identified as THSG substituted by a caffeyl group (162 Da, C_6_H_10_O_5_) and malonyl group (162 Da, C_6_H_10_O_5_) for the typical 162 Da and 86 Da neutral loss (Qiu et al., 2013; Liang et al., 2018). Compound **D31**, with a precursor ion at *m/z* 551.1559 (C_29_H_27_O_11_), was identified tentatively as THSG substituted by a coumaroyl group (146 Da, C_9_H_6_O_2_), according to the 146 Da neutral loss (Wang et al., 2017; Yang et al., 2019); specific information is shown in Supplementary Table S1.

### 3.3 Analysis of anthraquinones

Anthraquinones are the most important and controversial ingredients in PMR. They show high-profile hepatotoxicity and widely reported pharmacological (anti-inflammatory, anti-cancer, antidepressant, and resistance to microorganisms) activities (Lin et al., 2015; Yang et al., 2018; Campora et al., 2021; Yang et al., 2022). Anthraquinone compounds reported in PMR so far mainly include anthraquinone derivatives of different degrees of reduction and dimers, such as anthraquinone, anthrone, and dianthrone (Li and Jiang, 2018). On the basis of whether they combine with sugars, anthraquinones in PMR can also be divided into conjugated anthraquinones and free anthraquinones. In addition, in view of its nucleus, there is an emodin nucleus and a physcion nucleus in anthraquinones.

The peak of the emodin reference substance was detected at 29.889 min, and [M-H]^−^ was at *m/z* 269.0454 (C_15_H_9_O_5_). After crazing in the collision pool, a unit of CO (28 Da) was lost, and the characteristic fragment ion at *m/z* 241 (C_14_H_9_O_4_) was generated. With an increase in collision energy, the debris also lost 16 Da (O) to produce another characteristic fragment ion at *m/z* 225.0560 (C_14_H_9_O_3_). So, we believed that the typical fragments generated by anthraquinone compounds with emodin as the parent nucleus in MS were at *m/z* 269 (C_15_H_9_O_5_) and *m/z* 241 (C_14_H_9_O_4_) or *m/z* 240 (C_14_H_8_O_4,_ radical ions) and *m/z* 225 (C_14_H_9_O_3_) (Qiu et al., 2013). For example, the standard substances emodin-1-O-*β*-glucoside and emodin-8-O-glucoside were the isomers formed by combining glucose on the first hydroxy group and eighth hydroxy group of the emodin nucleus, which were eluted at 17.418 min and 21.949 min, respectively. Their precursor ions at *m/z* 431.0984 (C_21_H_19_O_10_) produced characteristic fragment ions at *m/z* 269 (C_15_H_9_O_5_) by breaking the glycosidic bond and losing one glucose unit (162 Da, C_6_H_10_O_5_) under the action of collision energy.

Another anthraquinone reference substance, physcion-8-O-glucopyranoside, was observed at 24.78 min upon MS, and its primary ion was at *m/z* 445.1128 (C_22_H_21_O_10_). Similar to emodin-1-O-*β*-glucoside, glycosidic bonds were broken, along with the loss of one unit of glucoside (162 Da, C_6_H_10_O_5_) and the generation of the characteristic fragment ion at *m/z* 283 (C_14_H_9_O_4_), matching with the excimer ion of physcion.

In addition, the reference substance *cis*-emodin–physcion dianthrone is a typical example to explain the cracking tendency of dianthrone compounds. It comprises a parent nucleus of emodin and physcion, with a retention time of 9.952 min and [M-H]^−^ at *m/z* 523.1397 (C_31_H_23_O_8_). After experiencing a certain amount of collision energy, the C10–C10’ bond connecting the two parent nuclei broke, and the characteristic fragment at *m/z* 254 (C_14_H_9_O_4_) was generated. In accordance with the literature, we assumed that dianthrones, such as emodin–physcion bianthrone, emodin–physcion bianthrone, and physcion–physcion bianthrone, tended to suffer breakage of the glycosidic bond and the C10–C10’ bond under the impact of collision energy (Yang et al., 2018).

By comparing the retention time and MS behavior with the reference substances, compounds **A7**, **A12**, **A17**, **A41**, and **A47** were determined to be emodin-1-O-glucoside, emodin-8-O-*β*-glucoside, physcion-8-O-glucopyranoside, emodin, and *cis*-emodin–physcion bianthrone, respectively.

According to the rules summarized by the previously stated reference substances, compounds **A6**, **A10**, **A13**, **A14**, **A15**, **A23**, and **A30** were speculated to be anthraquinones with a framework of emodin based on the characteristic fragment ion at *m/z* 269 (C_15_H_9_O_5_) or *m/z* 225 (C_14_H_9_O_3_). Taking compound **A14** as an example, its [M-H]^−^ ion was 517.0988 (C_24_H_21_O_13_), and it possessed the main fragment ions at *m/z* 473 (C_23_H_21_O_11_), *m/z* 431 (C_21_H_19_O_10_), and *m/z* 269 (C_15_H_9_O_5_), which were presumed to be generated after losing a molecule of CO_2_ (44 Da), a malonyl group (86 Da, C_3_H_2_O_3_), and one glucose unit (162 Da, C_6_H_10_O_5_) successively. The neutral loss of glucose (162 Da) occurred mainly after the shedding of a malonyl group (86 Da), so we speculated that the malonyl group was connected to glucose, and, by comparison with the literature, compound **A14** was identified as emodin-O-(malonyl)-glucopyranoside (Liang et al., 2018). In a similar way, first, compounds **A10** and **A13** were preliminarily speculated as emodin derivatives according to the characteristic fragment ion at *m/z* 269 (C_15_H_9_O_5_). On the other hand, they lost 44 Da and 42 Da neutral segments separately to produce diagnostic ions at *m/z* 269, by which we deduced that there was a carboxyl group and an acetyl group, respectively. Ultimately, compounds **A10** and **A13** were inferred as acetyl-emodin-glucopyranoside and endocrocin, respectively. The primary and secondary fragment ions of compound **A15** and compound **A10** were identical, except for different retention times, so we speculated them to be isomers of acetyl-emodin-glucopyranoside. As for compound **A6**, it presented a relatively rare neutral loss (80 Da, SO_3_) and the typical diagnostic ion of emodin derivatives (*m/z* 269). By comparing with the literature, we identified it as emodin-8-O-*β*-hexose-sulfate (Wang et al., 2017).

Compared to the aforementioned compounds, compounds **A9**, **A19**, **A23**, **A29**, and **A30** had a comparatively special way of cracking. As for compounds **A29** and **A30**, they both generated the main fragment ion at *m/z* 283, one of the diagnostic ions of physcion. Moreover, they were both more likely to lose 28 Da (CO) in the collision pool, which was also consistent with anthraquinones. By searching relevant literature, they were tentatively taken for emodin methyl ester and acetyl-emodin (Wang et al., 2017; Huang et al., 2018; Wang et al., 2021). In the same way, compound **A23** was speculated to be an isomer of acetyl emodin. Compared with compound **A29**, we could find that the molecular weight of compound A9 was 15 Da higher, but the neutral loss (28 Da and 15 Da) was the same as that of compound **A29**. So, we presumed that compound **A9** was composed of emodin methyl ester plus methylene and was mostly like questinol (Qiu et al., 2013). Similarly, the molecular weight of compound **A19** was 2 Da higher than that of compound **A29** and lost 28 Da and 16 Da in succession in the collision pool; we deduced that it could be an emodin methyl ester analog. We preliminarily speculated it as citreorosein (Huang et al., 2018).

Other atypical anthraquinone derivatives were also present in PMR. Compounds **A1** and **A3** were anthranones and produced a fragment ion at *m/z* 255 (C_14_H_10_O_4_) upon MS/MS. The primary ion of compound **A1** at *m/z* 479.1189 (C_22_H_23_O_12_) shattered into fragment ions at *m/z* 299 (C_16_H_11_O_6_) and *m/z* 255 (C_14_H_10_O_4_) by losing 180 Da (glucose and H_2_O) and 44 Da (CO_2_). As a result, compound **A1** was presumed to be a compound ion of emodin acid-hexose (Qiu et al., 2013). Compound **A3** had [M-H]^−^ at *m/z* 417.1195 (C_21_H_21_O_9_), which produced a characteristic fragment ion at *m/z* 255 (C_14_H_10_O_4_) by losing 162 Da (hexose) and was speculated to be cassialoin (Luo et al., 2017). The [M-H]^−^ of compound **A5** was at *m/z* 447.0933 (C_14_H_8_O_5_), and a fragment ion at *m/z* 285 (C_15_H_9_O_6_) was generated after 162 Da (hexose) had been lost in the collision pool, so we speculated it to be citreorosein-8-O-glucopyranoside (Wang et al., 2015).

In view of the relatively higher molecular weight, successive breakage of glycosidic bonds and characteristic fragment ions at *m/z* 254 and *m/z* 269, compounds **A2**, **A8**, **A11**, **A16**, **A18**, **A20**, **A22 A24–A28**, **A31–A40**, **A42–A45**, and **A48–A50** were speculated to be dianthranones (Yang et al., 2019), and information on specific compounds is provided in Supplementary Table S1.

### 3.4 Analysis of naphthalene compounds

Naphthalene compounds are important polyphenols in PMR. So far, three types of naphthalene compounds have been isolated from PMR: torachrysones (e.g., torachrysone-8-O-*β*-D-glucopyranoside), hydroxymusizins (e.g., 6-hydroxy-8-o-glucoside), and naphthoquinones (e.g., 2-methoxy-6-acetyl-7-methyljuglone) (Reddy. 2016; Luo et al., 2017; Yang et al., 2020). Different mother nucleus structures tend to generate different characteristic fragment ions, which guide the structural analysis of compounds.

The standard substances torachrysone-8-O-*β*-D-glucopyranoside and polygonimitin E were formed by combining one or two units of hexose, respectively, with a torachrysone skeleton. In LC-MS, torachrysone-8-O-*β*-D-glucopyranoside and polygonimitin E, with [M-H]^−^ at *m/z* 407.1346 (C_20_H_23_O_9_) and *m/z* 539.1770 (C_25_H_31_O_13_), were eluted successively at 19.2 min and 17.7 min, respectively. Under the effect of collision energy, they all broke the glycosidic bond and lost one or two units of hexose (162 Da, C_6_H_10_O_5_), resulting in the characteristic fragment ion at *m/z* 245 (C_14_H_13_O_4_). Then, the methyl radical was lost in rapid sequence to produce another characteristic ion at *m/z* 230 (C_13_H_10_O_4_).

Six naphthalene-based components were resolved, among which compounds **N4** and **N6** were determined to be polygonimitin E and torachrysone-8-O-glucoside, respectively, by comparing the excimer ions, fragment ions, and retention time. Compound **N5** was eluted at 19.1 min, with [M-H]^−^ at *m/z* 245.0821 (C_14_H_13_O_4_), which was 162 Da (C_6_H_10_O_5_) lower than torachrysone-8-O-*β*-glucoside. In addition, combining with the characteristic fragment ion at *m/z* 230 (C_13_H_10_O_4_), compound **N5** was speculated to be torachrysone. Similarly, the molecular weight of compound **N7** was 42-Da (C_2_H_2_O) greater than that of the reference substance torachrysone-8-O-*β*-glucoside. Moreover, it was easy to lose 42 Da (C_2_H_2_O) to generate a fragment ion at *m/z* 405 (C_20_H_21_O_9_) followed by *m/z* 230 in MS/MS. The 42 Da (C_2_H_2_O) could be regarded as acetyl substitution (C_2_H_2_O); therefore, compound **N7** was speculated to be torachrysone-8-O-(6′-O-acetyl)-D-glucopyranoside (Wang et al., 2017).

We found that hydroxymusizins and torachrysones differ only by one unit of methyl substitution (15 Da, CH_3_) and may have similar cleavage pathways and fragment ions. Taking compounds **N1** and **N3** as examples, the molecular weight of compounds **N3** and **N1** was 14 Da (CH_2_) lower than that of the reference substance torachrysone-8-O-glucoside, and the fragment ion at *m/z* 231 (C_13_H_11_O_4_) was generated after losing 162 Da (C_6_H_10_O_5_) in MS/MS. Therefore, we assumed that compounds **N1** and **N3** were hydroxymusizin-O-glucopyranoside and their isomers (Jin et al., 2007). Similarly, compound **N2** was presumed to be hydroxymusizin-o-glucose-o-xylose.

### 3.5 Analysis of other compounds

**FIGURE 10:**
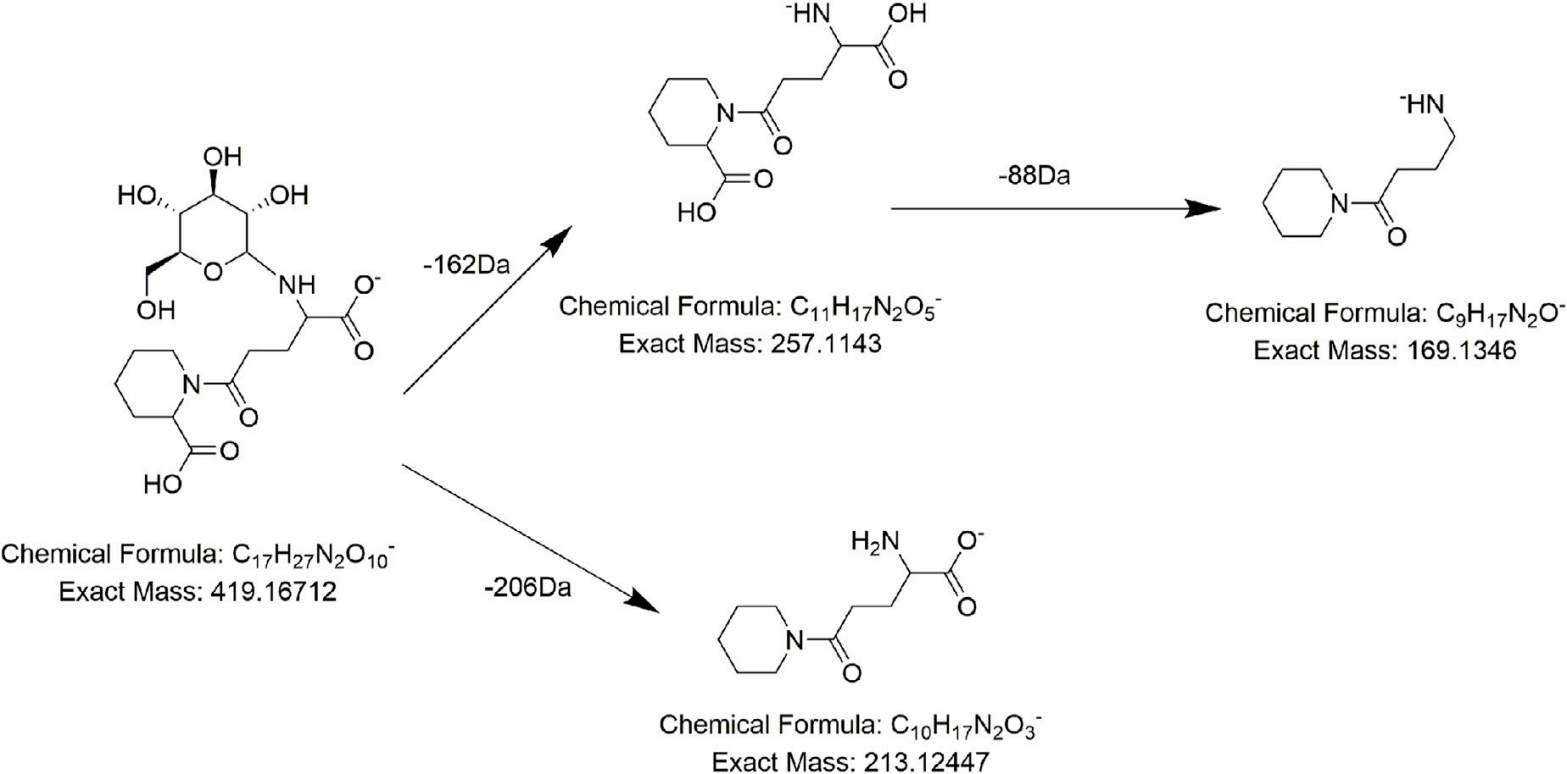
Proposed fragmentation pathways of **AM7** (gamma-L-glutamyl-L-pipecolic-acid glucopyranoside).

**FIGURE 11:**
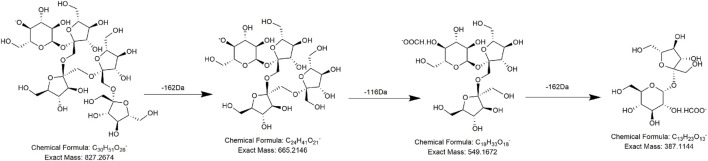
Proposed fragmentation pathways of **G4** (1F-fructofuranosylnystose).

**FIGURE 12:**
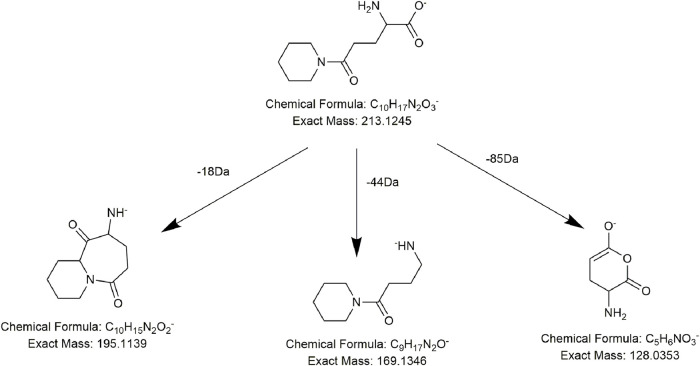
Proposed fragmentation pathways of **AM9** (1-piperidinepentanoic acid, α-amino-δ-oxo-, (S)-).

Alkaloids, fatty acids, phospholipids, amino acids, and sugars have also been found in PMR (Lin et al., 2015; Yang et al., 2020; Yang et al., 2021). We identified several compounds for the first time in PMR: three amino acids, two saccharides, and two fatty acids. Taking compound **AM7** as an example, it generated the main fragment ion at *m/z* 257.1143 after losing 162 Da (C_6_H_10_O_5_) upon MS. The main fragment ion at *m/z* 257.1143 and its additional debris was consistent with the MS behavior of the identified compound gamma-L-glutamyl-L-pipecolic-acid reported in the literature (Huang et al., 2022). Hence, **AM7** was thought to be gamma-L-glutamyl-L-pipecolic-acid glucopyranoside (Figure 10). Similarly, two saccharides, 1F-fructofuranosylnystose (**G4**, Figure 11) and maltose (**G5**), were deduced by the continuous loss of hexose molecules and the previously reported compounds raffinose and cephulac (Wang et al., 2017). Compound **AM9** was tentatively identified as 1-piperidinepentanoic acid, α-amino-δ-oxo-, (S)- by the TCM library of Agilent Technology, and the proposed cracking pathway is shown in Figure 12.

## 4 Discussion

In recent years, discussion of the hepatotoxicity of PMR has increased, and our research team has also explored the hepatotoxic components of PMR (Yang et al., 2018; Li et al., 2020; Gao et al., 2022; Song et al., 2022). We showed that solutions of PMR extracted with different solvents showed different percentages of hepatotoxicity, and the difference in toxicity is mostly caused by chemical differences in each solvent (Yang et al., 2018). A comprehensive identification and analysis of the chemical constituents of PMR are of far-reaching significance to clarify its material basis and toxic mechanism.

**FIGURE 13:**
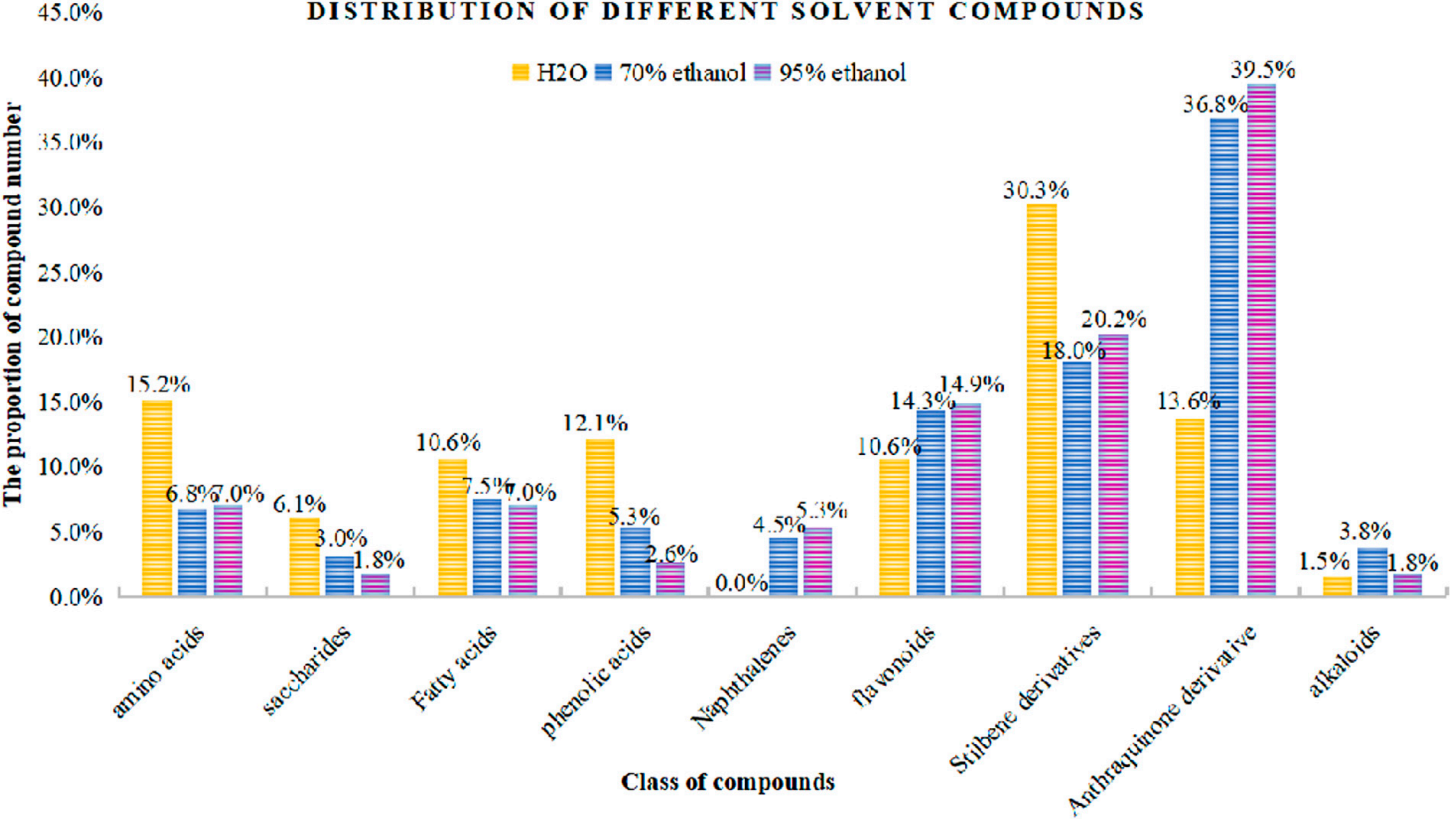
Distribution of compound amount in different solvents.

**FIGURE 14:**
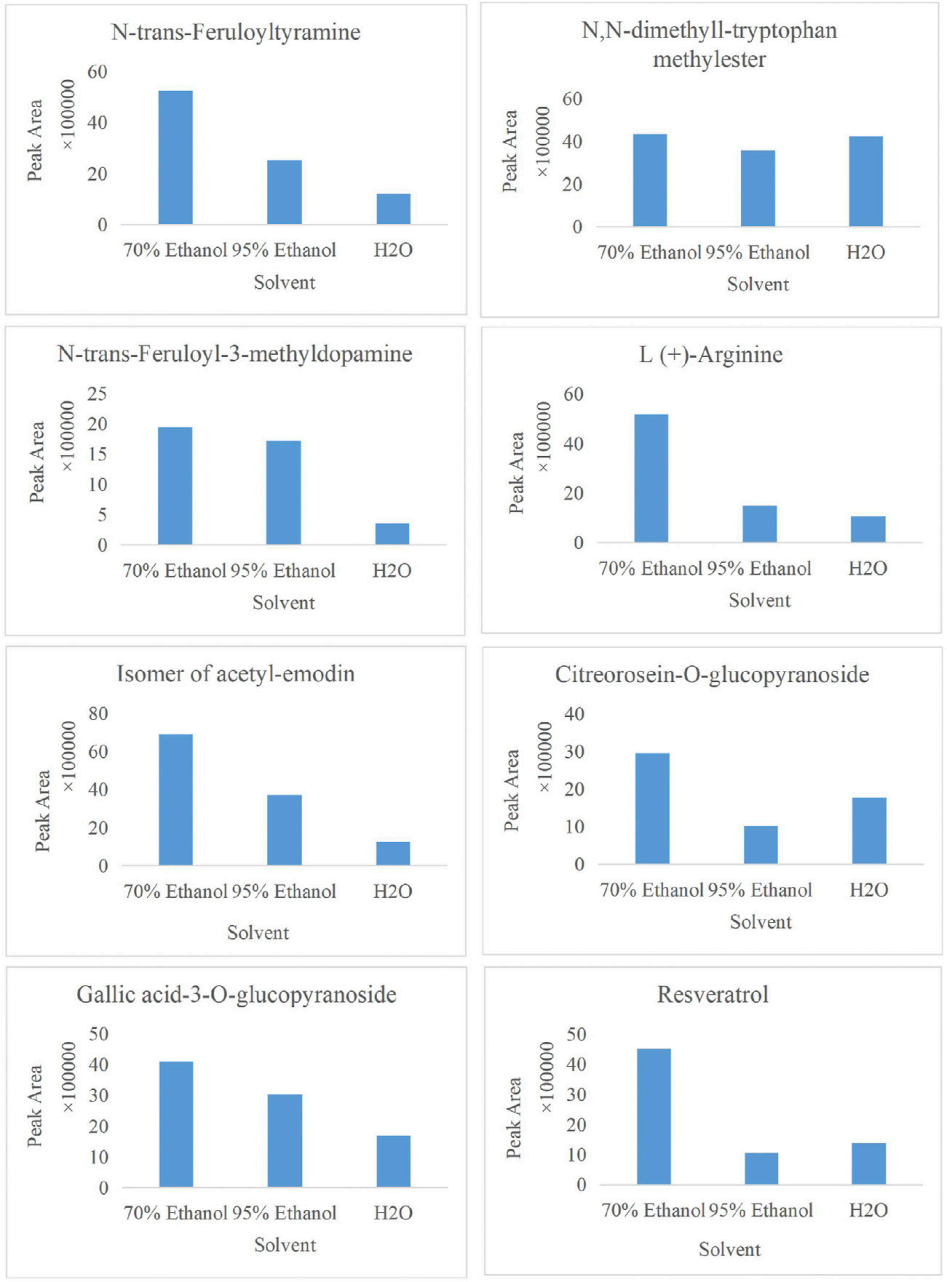
Histogram of peak area of compound N,N-dimethyl-tryptophan methylester, N-trans-feruloyltyramine, N-trans-feruloyl-3-methyldopamine, L (+)-arginine, the isomer of acetyl-emodin, citreorosein-O-glucopyranoside, gallic acid-3-O-glucopyranoside, and resveratrol in three solvents.

**FIGURE 15:**
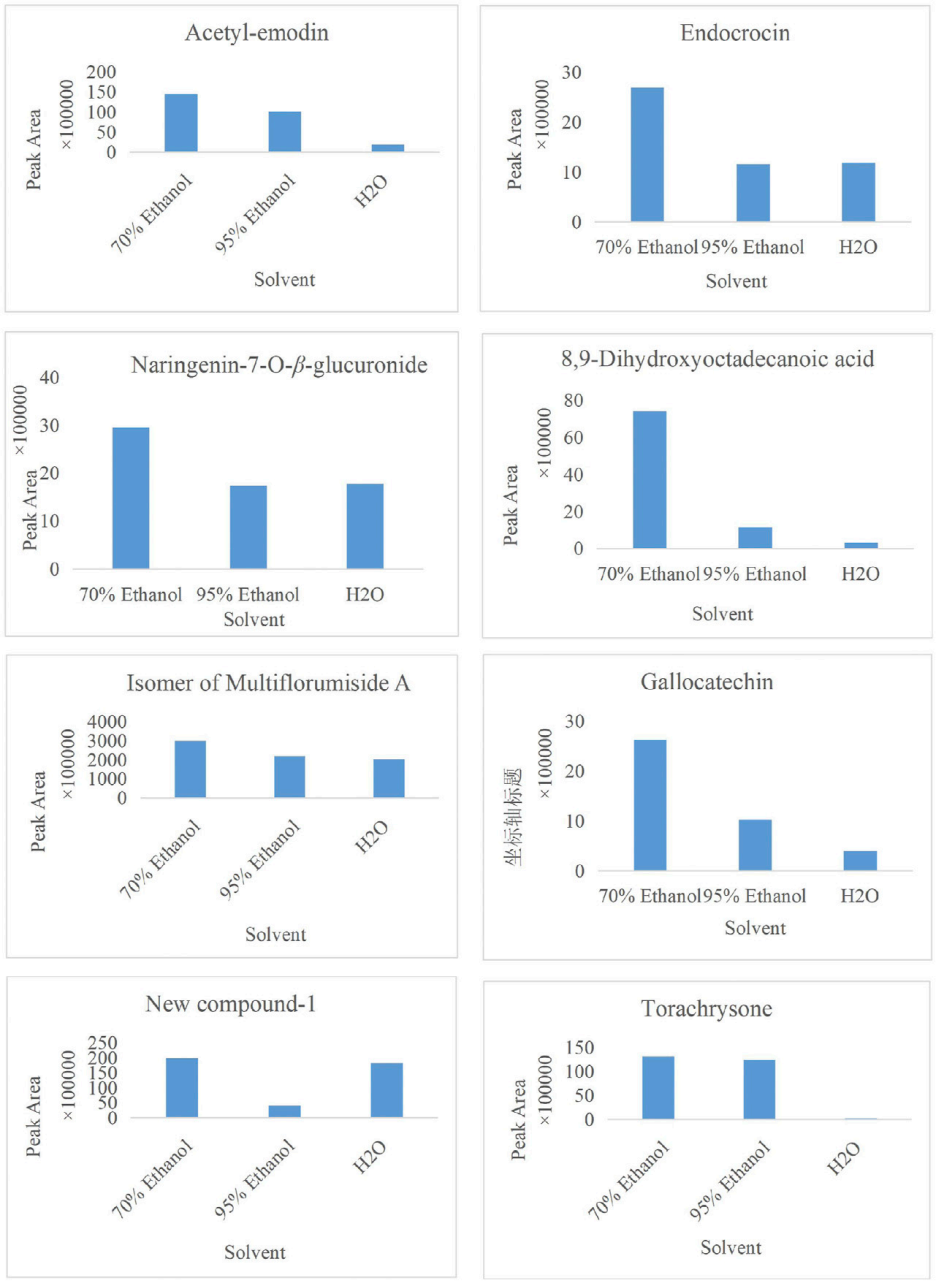
Histogram of peak area of compound acetyl-emodin, endocrocin, naringenin-7-O-β-glucuronide, 8,9-dihydroxyoctadecanoic acid, the isomer of multiflorumiside A, gallocatechin, new compound-1, and torachrysone in three solvents.

In the present study, a UPLC-Q-ToF-MS/MS method was established to systematically characterize and compare the components in three extracts of PMR. A total of 152 compounds were identified, of which 66, 133, and 114 compounds were identified in aqueous solution, 70% ethanol solution, and 95% ethanol solution, respectively. Simultaneously, we found that amino acids and stilbene derivatives were the most important compounds in the aqueous extract of PMR and that extracts in 70% ethanol and 95% ethanol contained more anthraquinones. Figure 13 shows the distribution of specific compounds. Moreover, 16 components show relatively higher content in the extract of 70% ethanol, including three alkaloids, one amino acid, four anthraquinones, one fatty acid, two polyphenols, and two stilbene derivatives. For these 16 compounds, the comparing histograms are shown in Figures 14,Figures 15 Figures 15 A statistical table of the peak area is attached in the Supplementary Material.

A few works of literature have reported the biological activity and toxicity of three alkaloidal constituents. (Ghosal et al. (1972) have reported that the total alkaloid fraction of *E. variegata*, including N,N-dimethyl-L-tryptophan methylester, has multiple effects: neuromuscular blocking, smooth muscle relaxant, central nervous system depressant, hydrocholeretic, and anticonvulsant effects. Regarding N-trans-feruloyltyramine, another alkaloid, there are some academic papers that demonstrate its antibacterial activity, *α*-glucosidase inhibitory activity, and antioxidant activity (Yokozawa et al., 2001; Panidthananon et al., 2018; Gao et al., 2019). However, its cytotoxicity is worth noting, which is normally involved in host defense mechanisms such as anti-bacterial (mainly G-bacteria), antiviral, and anti-parasitic but can destroy the body’s own cells, leading to tissue injury and disease in some pathological cases (Hsieh et al., 2001; Zhao et al., 2018; Gao et al., 2019). Similar to N-trans-feruloyltyramine, compound N-trans-feruloyl-3-methyldopamine also causes cytotoxicity (Zhao et al., 2018). Regarding the four anthraquinones, endocrocin, citreorosein-O-glucopyranoside, and acetyl-emodin and its isomer all belong to emodin derivatives, and the differences are the substituent groups that are attached. Endocrocin is mainly used as a pigment, and a small amount of literature has reported its biological activities, such as antifungal, antitumor, antioxidant, and pest-control effects (Liu et al., 2012; Wu, 2022). Zhang reported the neuroprotective effect of 2-acetyl-emodin, whose activity and toxicity need more exploration. As for citreorosein-O-glucopyranoside, the metabolites citreorosein and its isomer, aloe-emodin, have received more attention. Molecular docking experiment results showed that citreorosein has a strong binding ability with CYP1A1, CYP1A2, CYP2E1, CYP2B6, CYP2C9, and CYP3A4, and it is highly likely to induce liver toxicity by inhibiting CYP450 isoenzyme activity (Huang et al., 2021). Wang found that mild accumulation of aloe emodin and citreorosein was presented with the increase in dosage (Wang et al., 2022). Moreover, compared with the solvent control group, citreorosein 6.25, 12.5, and 25 μg·mL-1 groups significantly increased the mutation rate of the PIG-A gene *in vitro* (Wang et al., 2022). Thus, combined with our research results, more attention should be paid to citreorosein-O-glucopyranoside, which could be a potential hepatotoxic component. Gallocatechin and naringenin-7-O-β-glucuronide belong to flavonoids. Flavonoids are widely recognized as a group of active components that have anti-inflammatory, antioxidant, detoxification, hypoglycemic, and anticancer effects, and there have been no reports on their toxicity (Wang et al., 2019; Yuan et al., 2019; Dong et al., 2022). Similarly, resveratrol, as a compound that has been well-studied, processes many biological activities, including neuroprotective, antitumor, anti-inflammatory, and hepatoprotective effects (Yin et al., 2022; Ping, 2023; Yan et al., 2023). Multiflorumiside A, a dimer of tetrahydroxy stilbene glycoside, was first isolated and reported for its suppressive effects against NO production in lipopolysaccharide-stimulated RAW 264.7 cells (Li et al., 2018). More clinical value and risk assessment should be carried out.

These compounds deserve additional attention in further hepatotoxicity studies of PMR. Our study lays a solid foundation for the subsequent screening of toxicity and quality-control indicators of PMR. There are still some points in this study that need further improvement. On one hand, only one ionization mode was used to analyze the samples in our experiment, so some compounds that easily ionize hydroxide ions may not be adequately identified. On the other hand, more batches of samples are needed to verify the content distribution of 16 components in three types of solutions.

## Data Availability

The original contributions presented in the study are included in the article/Supplementary Material; further inquiries can be directed to the corresponding authors.
