# Supplementary material for: On-line identification of the chemical constituents of Polygoni Multiflori Radix by UHPLC-Q-ToF MS/MS
Source: Front Chem. 2023 Apr 21;11:1158717. doi: 10.3389/fchem.2023.1158717 (PMC10160465; doi:10.3389/fchem.2023.1158717)
Supplement: Supplementary file 1 [file DataSheet1.doc]

**Supplementary Material**

# On-line Identification of Chemical Constituents in Polygoni Multiflori Radix by UHPLC-Q-TOF MS/MS

Xueting Wang^†^, Jianbo Yang^†^, Xianlong Cheng, Huiyu Gao, Yunfei Song, Ying Wang, Feng Wei^,*^, Shuangcheng Ma ^*^

Institute for Control of Chinese Traditional Medicine and Ethnic Medicine, National Institutes for Food and Drug Control, Beijing, 100050, China

^†^ These authors contributed equally to this work.

* Corresponding authors:

Feng Wei：[weifeng@nifdc.org.cn](mailto:weifeng@nifdc.org.cn)

Shuangcheng Ma：[masc@nifdc.org.cn](mailto:masc@nifdc.org.cn)

1. **Supplementary data**

**Supplementary data 1.** Comparison of peak areas of 16 compounds

|  | Compound | classification | Peak area (70% Ethanol) | Peak area (95% Ethanol) | Peak area (H2O) |
| --- | --- | --- | --- | --- | --- |
| 1 | N,N-dimethyl-L-tryptophan methylester | Alkloids | 4350313 | 3562344 | 4231097 |
| 2 | N-trans-Feruloyltyramine | Alkloids | 5257666 | 2531592 | 1217483 |
| 3 | N-trans-Feruloyl-3-methyldopamine | Alkloids | 1939288 | 1715093 | 353881 |
| 4 | L (+)-Arginine | Amino acid | 5178909 | 1497077 | 1068679 |
| 5 | Citreorosein-O-glucopyranoside | Anthroquinone | 2955634 | 1020555 | 1775985 |
| 6 | Endocrocin | Anthroquinone | 2688466 | 1158702 | 1180154 |
| 7 | Isomer of acetyl-emodin | Anthroquinone | 6898133 | 3736828 | 1269264 |
| 8 | Acetyl-emodin | Anthroquinone | 14454716 | 10130545 | 1839633 |
| 9 | 8,9-Dihydroxyoctadecanoic acid | Fatty acid | 7416048 | 1134756 | 314925 |
| 10 | Gallic acid-3-O-glucopyranoside | Fatty acid | 4102916 | 3016492 | 1700754 |
| 11 | Gallocatechin | flovonoids | 2621483 | 1014924 | 401584 |
| 12 | naringenin-7-O-β-glucuronide | flovonoids | 2955634 | 1744077 | 1775985 |
| 13 | torachrysone | Polyphenols | 13138950 | 12404004 | 82253 |
| 14 | New compound-1 | Polyphenols | 19784778 | 3980932 | 18221235 |
| 15 | Isomer of Multiflorumiside A | Stilbene derivatives | 297892726 | 219761889 | 203118762 |
| 16 | Resveratrol | Stilbene derivatives | 4504602 | 1060665 | 1389297 |

**Supplementary data 2. Mass data of compounds identified from *Polygonum Multiflorum* Thunb.**

| peak | No. | t_R_  (min) | identification | Proposed formula [M-H]^-^ | Measured Mass [M-H]^-^ | Accuracy Mass [M-H]^-^ | Error (ppm) | MS/MS | H_2_O | 70%  EtOH | 95%  EtOH | classification | Ref |
| --- | --- | --- | --- | --- | --- | --- | --- | --- | --- | --- | --- | --- | --- |
| 1 | AM1 | 2.47 | L (+)-Arginine | C_6_H_13_N_4_O_2_^-^ | 173.1042 | 173.1044 | 1.2 | 158.9255;  131.0822[M-H-C_5_H_11_N_2_O_2_]^-^;  102.0560[M-H-C_4_H_8_NO_2_]^-^ | - | + | - | Amino acids | (Luo et al., 2017) |
| 2 | AM2 | 3.95 | L-Pyroglutamic acid | C_5_H_6_NO_3_^-^ | 128.0353 | 128.0353 | 0.0 |  | + | - | + | Amino acids | (Lin et al., 2015) |
| 3 | AM3 | 4.51 | Isomer of L-pyroglutamic acid | C_5_H_6_NO_3_^-^ | 188.0565[M-H+CH_3_COOH] | 188.0564 | 0.5 | 128.0353[M-H]^-^；  111.0088[M-H-NH_3_]^-^； | + | - | - | Amino acids | (Lin et al., 2015) |
| 4 | AM4 | 4.93 | L-Tyrosine | C_9_H_10_NO_3_^-^ | 180.0665 | 180.0667 | 1.1 | 163.0398[M-H-NH_3_]^-^; | + | - | - | Amino acids | (Wang et al., 2021) |
| 5 | AM5 | 5.78 | Isomer of gamma-L-Glutamyl-L-pipecolic-acid | C_11_H_17_N_2_O_5_^-^ | 257.1143 | 257.1143 | 0.0 | 213.1240[M-H-CO_2_]^-^;  195.1135[M-H-CO_2_-H_2_O]^-^;  128.0355[M-H-CO_2_-C_5_H_11_N]^-^; | - | + | + | Amino acids | (Luo et al., 2017) |
| 6 | AM6 | 6.16 | Isomer of gamma-L-Glutamyl-L-pipecolic-acid | C_11_H_17_N_2_O_5_^-^ | 257.1142 | 257.1143 | 0.4 | 213.1240[M-H-CO_2_]^-^;  195.1135[M-H-CO_2_-H_2_O]^-^;  128.0355[M-H-CO_2_-C_5_H_11_N]^-^; | + | + | + | Amino acids | (Luo et al., 2017) |
| 7 | AM7^b^ | 6.28 | gamma-L-Glutamyl-L-pipecolic-acid glucopyranoside | C_17_H_27_N_2_O_10_^-^ | 419.1669 | 419.1671 | 0.5 | 257.1143[M-H-C_6_H_10_O_5_]^-^;  169.0145[M-H-C_6_H_10_O_5_-C_2_O_4_]^-^ | + | + | - | Amino acids | (Luo et al., 2017) |
| 8 | AM8 | 7.52 | gamma-L-Glutamyl-L-pipecolic-acid | C_11_H_17_N_2_O_5_^-^ | 257.1142 | 257.1143 | 0.4 | 213.1240[M-H-CO_2_]^-^;  195.1135[M-H-CO_2_-H_2_O]^-^;  128.0355[M-H-CO_2_-C_5_H_11_N]^-^; | + | + | + | Amino acids | (Luo et al., 2017) |
| 9 | AM9^b^ | 7.64 | 1-Piperidinepentanoic acid, *α*-amino-*δ*-oxo-, (S)- | C_10_H_17_N_2_O_3_^-^ | 213.1244 | 213.1245 | 0.5 | 195.1135[M-H-CO_2_-H_2_O]^-^;  128.0355[M-H-CO_2_-C_5_H_11_N]^-^; | + | + | + | Amino acids | (Luo et al., 2017) |
| 10 | AM10^b^ | 7.87 | Isomer of gamma-L-Glutamyl-L-pipecolic-acid glucopyranoside | C_17_H_27_N_2_O_10_^-^ | 419.1669 | 419.1671 | 0.5 | 257.1143[M-H-C_6_H_10_O_5_]^-^;  213.1240[M-H-CO_2_]^-^; | + | + | + | Amino acids | (Luo et al., 2017) |
| 11 | AM11 | 8.34 | 2-vynyl-1H-indole-carboxylic acid | C_11_H_8_NO_2_^-^ | 186.0560 | 186.0561 | 0.5 | 142.0661[M-H-CO_2_]^-^ | + | + | + | Amino acids | (Wang et al., 2017) |
| 12 | AM12 | 8.93 | Isomer of N-L-Glutamate-L-leucine | C_11_H_19_N_2_O_5_^-^ | 259.1299 | 259.1300 | 0.4 | 241.1200[M-H-H_2_O]^-^；  223.1081[M-H-H_2_O]^-^； | + | + | + | Amino acids | (Zhao et al., 2020) |
| 13 | G1 | 2.87 | Raffinose | C_18_H_31_O_16_^-^ | 549.1668[M-H+HCOOH] | 549.1672 | 1.2 | 503.1623[M-H]^-^；  179.0563[M-H_2_O-C_6_H_10_O_5_-C_6_H_10_O_5_]^-^ | + | - | - | Saccharides | (Lin et al., 2015) |
| 14 | G2 | 2.94 | Cephulac | C_12_H_22_O_11_^-^ | 387.1141[M-H+HCOOH]- | 387.1144 | 1.1 | 341.1088[M-H]^-^;  179.0558[M-H-C_6_H_10_O_5_]^-^ | + | + | + | Saccharides | (Wang et al., 2017) |
| 15 | G3^b^ | 2.99 | Maltose | C_24_H_41_O_21_^-^ | 665.2144 | 665.2146 | 0.0 | 485.1505[M-H_2_O-C_6_H_10_O_5_]^-^；  341.1086[M-H_2_O-C_6_H_10_O_5_]^-^；  179.0564[M-H_2_O-C_6_H_10_O_5_-C_6_H_8_O_4_]^-^ | + | + | - | Saccharides | (Lin et al., 2015) |
| 16 | G4^b^ | 3.10 | 1F-Fructofuranosylnystose | C_30_H_51_O_26_^-^ | 827.2607 | 827.2674 | 0.4 | 665.2147[M-H-C_6_H_10_O_5_]^-^；  549.1666[M-H-C_12_H_20_O_10_+HCOOH]^-^；  387.1144[M-H-C_18_H_20_O_15_+HCOOH]^-^ | + | - | - | Saccharides | (Wang et al., 2017) |
| 17 | G5 | 3.51 | sucrose | C_12_H_22_O_11_^-^ | 387.1143[M-H+HCOOH]^-^ | 387.1144 | 0.5 | 341.1091[M-H]^-^;  179.0563[M-H-C_6_H_10_O_5_]^-^ | - | + | - | Saccharides | (Huang et al., 2018) |
| 18 | F1^b^ | 3.11 | Shikimic acid | C_7_H_9_O_5_^-^ | 173.0455 | 173.0456 | 0.6 | 137.0238[M-H_4_O_2_]^-^ | - | - | + | Fatty acids | (Avula et al., 2009) |
| 19 | F2 | 3.22 | Hydroxysuccinic acid | C_4_H_6_O_5_^-^ | 133.0142 | 133.0142 | 0.0 | 115.0036[M-H_2_O]^-^ | + | + | + | Fatty acids | (Wang et al., 2017) |
| 20 | F3 | 3.46 | Isomer of Hydroxysuccinic acid | C_4_H_6_O_5_^-^ | 133.0142 | 133.0142 | 0.0 | 151.0037[M-H_2_O]^-^ | + | - | - | Fatty acids | (Wang et al., 2017) |
| 21 | F4 | 3.64 | Isomer of Hydroxysuccinic acid | C_4_H_6_O_5_^-^ | 133.0142 | 133.0142 | 0.0 | 151.0038[M-H_2_O]^-^ | + | - | - | Fatty acids | (Wang et al., 2017) |
| 22 | F5 | 3.93 | Hydocerol A | C_6_H_7_O_7_^-^ | 191.0198 | 191.0197 | 0.5 | 173.0093[M-H-H_2_O]^-^;  111.0088[M-H-H_2_O-CO_2_]^-^ | + | + | - | Fatty acids | (Wang et al., 2017) |
| 23 | F6 | 4.56 | Isomer of Hydocerol A | C_6_H_7_O_7_^-^ | 191.0198 | 191.0197 | 0.5 | 173.0082[M-H-H_2_O]^-^;  111.0084[M-H-H_2_O-CO_2_]^-^ | + | - | - | Fatty acids | (Wang et al., 2017) |
| 24 | F7 | 5.08 | Butanedioic acid | C_4_H_5_O_4_^-^ | 117.0192 | 117.0193 | 0.9 |  | + | + | - | Fatty acids | (Liu et al., 2009) |
| 25 | F8 | 25.55 | 9,12,13-TriHOME | C_18_H_33_O_5_^-^ | 329.2335 | 329.2334 | 0.3 | 311.2227[M-H-H_2_O]^-^;  293.2123[M-H-H_2_O-H_2_O]^-^;  229.1444[M-H-C_6_H_12_O]^-^;  211.1342[M-H-C_6_H_12_O-H_2_O]^-^; | + | + | + | Fatty acids | (Huang et al., 2021) |
| 26 | F9^b^ | 30.32 | 8,9-Dihydrox  yoctadecanoic acid | C_17_H_33_O_2_^-^ | 315.2536 | 315.2540 | 1.3 | 297.2439[M-H-H_2_O]^-^; | - | + | - | Fatty acids | (Wheelan et al., 1995) |
| 27 | F10 | 37.41 | Pentadecanal | C_16_H_31_O_3_^-^ | 271.2279[M-H+HCOOH]^-^ | 271.2279 | 0.0 | 225.2224[M-H]^-^ | - | + | + | Fatty acids | (Huang et al., 2021) |
| 28 | F11 | 40.82 | Linoleic acid339.23 | C_18_H_31_O_2_^-^ | 279.2332 | 279.2330 | 0.7 | 261.2222[M-H-H_2_O]^-^； | - | + | + | Fatty acids | (Rui 2018) |
| 29 | F12 | 44.00 | (9Z,12Z)-n-hexadecanoic acid | C_16_H_31_O_2_^-^ | 255.2329 | 255.2332 | 1.2 |  | - | + | + | Fatty acids | (Zhang 2016) |
| 30 | F13 | 45.29 | 9-octadecenoic acid | C_18_H_33_O_2_^-^ | 281.2487 | 281.2486 | 0.4 |  | - | + | + | Fatty acids | (Zhang 2016) |
| 31 | P1 | 3.43 | Gallic acid-3-O-glucopyranoside | C_13_H_15_O_10_^-^ | 331.0669 | 331.0670 | 0.3 | 169.0144[M-H-C_6_H_10_O_5_]^-^ | - | + | - | Polyphenols | (Luo et al., 2017) |
| 32 | P2 | 5.83 | Isomer of Gallic acid-3-O-glucopyranoside | C_13_H_15_O_10_^-^ | 331.0667 | 331.0670 | 0.9 | 169.0144[M-H-C_6_H_10_O_5_]^-^ | + | - | - | Polyphenols | (Luo et al., 2017) |
| 33 | P3 | 6.11 | Isomer of Gallic acid-3-O-glucopyranoside | C_13_H_15_O_10_^-^ | 331.0667 | 331.0670 | 0.9 | 169.0144[M-H-C_6_H_10_O_5_]^-^ | + | - | - | Polyphenols | (Luo et al., 2017) |
| 34 | P4^a^ | 6.46 | Gallic acid | C_7_H_5_O_5_^-^ | 169.0146 | 169.0142 | 2.4 | 125.0244[M-H-CO_2_]^-^;  107.0134[M-H-CO_2_-H_2_O]^-^ | + | + | + | Polyphenols | / |
| 35 | P5 | 8.10 | Protocatechuic acid | C_7_H_5_O_4_^-^ | 153.0193 | 153.0193 | 0.0 | 109.0293[M-H-CO_2_]^-^ | + | - | - | Polyphenols | (Wang et al., 2021) |
| 36 | P6 | 9.04 | 3,4-Dihydroxybenzaldehyde | C_7_H_5_O_3_^-^ | 137.0244 | 137.0244 | 0.0 | 109.0309[M-H-CO]^-^ | + | - | + | Polyphenols | (Wang et al., 2021) |
| 37 | P7 | 12.10 | Coumaric acid | C_9_H_7_O_3_^-^ | 163.0399 | 163.0401 | 1.2 | 119.0506[M-H-CO_2_]^-^ | + | + | - | Polyphenols | (Luo et al., 2017) |
| 38 | N1 | 9.41 | Isomer of hydroxymusizin-O-glucopyranoside | C_19_H_21_O_9_^-^ | 393.1188 | 393.1191 | 0.8 | 231.0666[M-H-C_6_H_10_O_5_]^-^ | - | - | + | Naphthalenes | (Jin et al., 2007) |
| 39 | N2 | 13.64 | hydroxymusizin-O-glucose-O-xylose | C_24_H_29_O_13_^-^ | 525.1611 | 525.1614 | 0.6 | 363.0881[M-H-C_6_H_10_O_5_]^-^；  231.0658[M-H-C_6_H_10_O_5_-C_5_H_8_O_4_]^-^ | - | + | + | Naphthalenes | (Jin et al., 2007) |
| 40 | N3 | 14.57 | hydroxymusizin-O-glucopyranoside | C_19_H_21_O_9_^-^ | 393.1188 | 393.1191 | 0.8 | 259.1495[M-H-C_6_H_8_O_4_]^-^；  231.0663[M-H-C_6_H_10_O_5_]^-^ | - | + | + | Naphthalenes | (Jin et al., 2007) |
| 41 | N4^a^ | 17.75 | Polygonimitin E | C_25_H_31_O_13_^-^ | 539.1769 | 539.1770 | 0.2 | 245.0819[M-H-C_11_H_18_O_9_]^-^;  230.0583[M-H-C_11_H_18_O_9_-CH_3_]^-^ | - | + | + | Naphthalenes | / |
| 42 | N5 | 19.10 | torachrysone | C_14_H_13_O_4_^-^ | 245.0821 | 245.0819 | 0.8 | 230.0584[M-H-CH_3_]^-^ | - | + | - | Naphthalenes | (Wang et al., 2017) |
| 43 | N6^a^ | 19.16 | torachrysone-8-O-glucoside | C_20_H_23_O_9_^-^ | 407.1349 | 407.1348 | 0.2 | 245.0822[M-H-C_6_H_10_O_5_]^-^;  230.0587[M-H-C_6_H_10_O_5_-CH_3_]^-^ | - | + | + | Naphthalenes | / |
| 44 | N7 | 21.91 | torachrysone-8-O-(6’-O-acetyl)-D-glucopyranoside | C_22_H_25_O_10_^-^ | 449.1451 | 449.1453 | 0.4 | 407.1348245.0817[M-H-C_2_H_2_O]-  245.0817[M-H-C_2_H_2_O-C_6_H_10_O_5_]-;  230.0574[M-H-C_6_H_10_O_5_-CH_3_]^-^ | - | + | + | Naphthalenes | (Wang et al., 2017) |
| 45 | FL1 | 7.05 | Gallocatechin | C_15_H_133_O_7_^-^ | 305.0666 | 305.0667 | 0.3 | 153.0193[M-H-C_5_H_6_O_3_]^-^；  125.0242[M-H-C_9_H_8_O_4_]^-^ | - | + | - | Flavonoids | (Wang et al., 2017) |
| 46 | FL2 | 7.32 | Procyanidin B | C_30_H_25_O_12_^-^ | 577.1349 | 577.1351 | 0.3 | 425.0874[M-H-C_8_H_8_O_3_]^-^；  289.0715[M-H-C_15_H_12_O_6_]^-^；  125.0247[M-H-C_15_H_12_O_6_-C_9_H_8_O_3_]^-^ | - | + | + | Flavonoids | (Wang et al., 2017) |
| 47 | FL3 | 7.33 | Procyanidin C1 | C_45_H_37_O_18_^-^ | 865.1977 | 865.1985 | 0.9 | 713.1522[M-H-C_8_H_8_O_3_]^-^;  577.1343[M-H-C_15_H_12_O_6_]^-^;  125.0239[M-H-C_30_H_25_O_12_-C_9_H_8_O_3_]^-^ | + | - | - | Flavonoids | (Zhao et al., 2021) |
| 48 | FL4^n^ | 7.70 | New compound-2 | C_24_H_23_O_13_^-^ | 519.1121 | 519.1144 | 4.4 | 405.1203[M-H-C_4_H_2_O_4_]^-^；  243.0665[M-H-C_4_H_2_O_4_-C_6_H_10_O_5_]^-^ | - | + | + | Flavonoids | / |
| 49 | FL5 | 8.11 | mono-O-galloyl-procyanidin B | C_37_H_29_O_16_^-^ | 729.1454 | 729.1461 | 1.0 | 577.1349[M-H-C_7_H_4_O_4_]^-^;  407.0776[M-H-C_7_H_4_O_4_-C_8_H_10_O_4_]^-^;  289.0709[M-H-C_22_H_16_O_10_]^-^;  169.0138[M-H-C_30_H_24_O_11_]^-^;  125.0242[M-H-C_30_H_24_O_11_-CO_2_]^-^ | - | + | + | Flavonoids | (Qiu et al., 2013) |
| 50 | FL6^a^ | 8.46 | Catechin | C_15_H_13_O_6_^-^ | 289.0717 | 289.0718 | 0.3 | 245.0816[M-H-CO_2_]^-^;  151.0395[M-H-C_6_H_6_O_2_-CO]^-^;  125.0246[M-H-C_9_H_8_O_3_]^-^;  109.0293[M-H-C_9_H_8_O_3_-O]^-^; | + | + | + | Flavonoids | / |
| 51 | FL7 | 8.56 | Isomer of mono-O-galloyl-procyanidin B | C_37_H_29_O_16_^-^ | 729.1458 | 729.1448 | -1.4 | 577.1342[M-H-C_7_H_4_O_4_]^-^；  407.0767[M-H-C_7_H_4_O_4_-C_8_H_10_O_4_]^-^;  289.0713[M-H-C_22_H_16_O_10_]^-^；  169.0139[M-H-C_30_H_24_O_11_]^-^；  125.0250[M-H-C_30_H_24_O_11_-CO_2_]^-^； | - | + | + | Flavonoids | (Qiu et al., 2013) |
| 52 | FL8 | 8.70 | Isomer of procyanidin C1 | C_45_H_37_O_18_^-^ | 865.1977 | 865.1985 | 0.9 | 713.1435[M-H-C_8_H_8_O_3_]^-^;  577.1383[M-H-C_15_H_12_O_6_]^-^;  407.0758[M-H-C_15_H_12_O_6_-C_8_H_10_O_4_]^-^;  289.0727[M-H-C_30_H_24_O_12_]^-^;  125.0242[M-H-C_30_H_24_O_12_-C_9_H_8_O_3_]^-^ | + | + | + | Flavonoids | (Luo et al., 2017) |
| 53 | FL9 | 8.75 | Di-galloyl-procyanidin B | C_44_H_33_O_20_^-^ | 881.1571 | 881.1574 | 0.3 | 729.1441[M-H-C_7_H_4_O_4_]^-^;  559.1245[M-H-C_7_H_4_O_4_-C_8_H_6_O_5_]^-^;  407.0777[M-H-C_7_H_4_O_4_-C_8_H_6_O_5_-C_8_H_8_O_3_]^-^;  169.0140[M-H-C_37_H_28_O_15_]^-^ | - | + | + | Flavonoids | (Qiu et al., 2013) |
| 54 | FL10^a^ | 9.45 | Epicatechin | C_15_H_13_O_6_^-^ | 289.0717 | 289.0718 | 0.3 | 245.0820[M-H-CO_2_]^-^;  203.0712[M-H-CO_2_-C_2_H_2_O]^-^;  179.0348[M-H-C_6_H_6_O_2_]^-^;  151.0402[M-H-C_6_H_6_O_2_-CO]^-^;  125.0244[M-H-C_9_H_8_O_3_]^-^;  109.0294[M-H-C_9_H_8_O_3_-O]^-^; | + | + | + | Flavonoids | / |
| 55 | FL11 | 9.75 | Dihydrokaempferol-3-glucoside | C_21_H_21_O_11_^-^ | 449.1085 | 449.1089 | 0.9 | 403.1600[M-H-CH_2_O_2_]^-^；  287.0556[M-H-C_6_H_10_O_5_]^-^；  269.0483[M-H-C_6_H_10_O_5_-H_2_O]^-^；  243.0668[M-H-C_6_H_10_O_5_-H_2_O-CO_2_]^-^ | + | + | + | Flavonoids | (Huang et al., 2018) |
| 56 | FL12^n^ | 9.80 | Isomer of  new compound-2 | C_24_H_23_O_13_^-^ | 519.1121 | 519.1144 | 4.4 | 405.1203[M-H-C_4_H_2_O_4_]^-^；  243.0665[M-H-C_4_H_2_O_4_-C_6_H_10_O_5_]^-^ | - | + | + | Flavonoids | / |
| 57 | FL13^n^ | 10.10 | Isomer of  new compound-2 | C_24_H_23_O_13_^-^ | 519.1121 | 519.1144 | 4.4 | 405.1203[M-H-C_4_H_2_O_4_]^-^；  243.0665[M-H-C_4_H_2_O_4_-C_6_H_10_O_5_]^-^ | - | + | + | Flavonoids | / |
| 58 | FL14 | 10.28 | Catechin gallyl ester | C_22_H_17_O_10_^-^ | 441.0829 | 441.0827 | -0.5 | 289.0715[M-H-C_7_H_4_O_4_]^-^；  169.0141[M-H-C_15_H_12_O_5_]^-^；  125.0242[M-H-C_7_H_4_O_4_-C_9_H_8_O_3_]^-^ | - | + | + | Flavonoids | (Wang et al., 2021) |
| 59 | FL15^n^ | 10.35 | New compound-3 | C_24_H_19_O_14_^-^ | 555.0756 | 555.0780 | 4.3 | 511.0865[M-H-CO_2_]^-^；  441.0825[M-H-CO_2_-C_3_H_2_O_2_]^-^;  289.0716[M-H-CO_2_-C_3_H_2_O_2_-C_7_H_4_O_4_]^-^;  169.0142[M-H-C_19_H_14_O_9_]^-^;  125.0236[M-H-C_19_H_14_O_9_-CO_2_]^-^ | - | - | + | Flavonoids | / |
| 60 | FL16 | 10.82 | Polygonflavanol A | C_35_H_33_O_15_^-^ | 693.1824 | 693.1825 | 0.1 | 405.1193[M-H-C_6_H_10_O_5_]^-^;  289.0720[M-H-C_6_H_10_O_5_-C_14_H_10_O_4_]^-^; | - | + | + | Flavonoids | (Huang et al., 2018) |
| 61 | FL17^n^ | 11.50 | Isomer of  new compound-2 | C_24_H_23_O_13_^-^ | 519.1121 | 519.1144 | 4.4 | 405.1203[M-H-C_4_H_2_O_4_]^-^；  243.0665[M-H-C_4_H_2_O_4_-C_6_H_10_O_5_]^-^ | - | + | + | Flavonoids | / |
| 62 | FL18^a^ | 13.21 | Isomer of quercetin | C_15_H_9_O_7_^-^ | 301.0354 | 301.0354 | 0.0 | 257.0455[M-H-CO_2_]^-^;  151.0401[M-H-C_6_H_2_O_2_]^-^ | + | + | + | Flavonoids | / |
| 63 | FL19 | 16.12 | naringenin-7-O-*β*-glucuronide | C_21_H_19_O_11_^-^ | 447.0932 | 447.0933 | 0.2 | 271.0610[M-H-C_6_H_8_O_6_]^-^;  113.0243[M-H-C_10_H_7_O_2_-C_6_H_8_O_6_]^-^ | - | + | - | Flavonoids | (Rui et al., 2020) |
| 64 | FL20^b^ | 16.80 | Baicalin | C_21_H_17_O_11_^-^ | 445.0772 | 445.0776 | 0.9 | 269.0453[M-H-C_6_H_9_O_6_]^-^;  175.0253[M-H-C_15_H_11_O_5_]^-^;  113.0243[M-H-C_15_H_11_O_5_-CH_2_O_3_]^-^ | + | + | - | Flavonoids | (Wu et al., 2005) |
| 65 | FL21^n^ | 19.10 | New compound-4 | C_24_H_25_O_13_^-^ | 521.1279 | 521.1300 | 4.0 | 407.1346[M-H-C_4_H_2_O_4_]^-^；  245.0820[M-H-C_4_H_2_O_4_-C_6_H_10_O_5_]^-^ | - | + | + | Flavonoids | / |
| 66 | D1^n^ | 5.79 | New compound-1 | C_20_H_23_O_11_^-^ | 439.1243 | 439.1246 | 0.7 | 421.1140[M-H-H_2_O]^-^；  277.0720[M-H-C_6_H_10_O_5_]^-^;  259.0614[M-H--H_2_O-C_6_H_10_O_5_]^-^；  183.0305[M-H--H_2_O-C_6_H_10_O_5_-C_6_H_4_]^-^ | - | + | - | Polyphenols | / |
| 67 | D2^n^ | 6.04 | Isomer of  New compound-1 | C_20_H_23_O_11_^-^ | 439.1244 | 439.1246 | 0.5 | 421.1137[M-H-H_2_O]^-^；  277.0719[M-H-C_6_H_10_O_5_]^-^;  259.0615[M-H--H_2_O-C_6_H_10_O_5_]^-^；  183.0299[M-H--H_2_O-C_6_H_10_O_5_-C_6_H_4_]^-^ | + | + | - | Polyphenols | / |
| 68 | D3 | 6.87 | Polygonumoside C | C_40_H_43_O_19_^-^ | 827.2396 | 827.2404 | 1.0 | 665.1862[M-H-C_6_H_10_O_5_]^-^;  647.1813M-H-C_6_H_10_O_5_-H_2_O]^-^; | + | - | - | Stilbene derivatives | (Wang et al., 2017) |
| 69 | D4 | 7.27 | Polygonumoside D | C_40_H_43_O_19_^-^ | 827.2396 | 827.2404 | 1.0 | 665.1847[M-H-C_6_H_10_O_5_]^-^;  647.1800M-H-C_6_H_10_O_5_-H_2_O]^-^; | - | - | + | Stilbene derivatives | (Wang et al., 2017) |
| 70 | D5 | 7.40 | Isomer of Polygonumoside C/D | C_40_H_43_O_19_^-^ | 827.2396 | 827.2404 | 1.0 | 665.1878[M-H-C_6_H_10_O_5_]^-^;  647.1739[M-H-C_6_H_10_O_5_-H_2_O]^-^;  485.1235[M-H-C_6_H_10_O_5_-H_2_O-C_6_H_10_O_5_]^-^; | + | - | + | Stilbene derivatives | (Wang et al., 2017) |
| 71 | D6 | 7.67 | Isomer of Polygonumoside C/D | C_40_H_43_O_19_^-^ | 827.2396 | 827.2404 | 1.0 | 665.1841[M-H-C_6_H_10_O_5_]^-^;  647.1754[M-H-C_6_H_10_O_5_-H_2_O]^-^; | + | + | - | Stilbene derivatives | (Wang et al., 2017) |
| 72 | D7^a^ | 7.86 | Multiflorumiside A | C_40_H_43_O_18_^-^ | 811.2488 | 811.2455 | -4.1 | 649.1901[M-H-C_6_H_10_O_5_]-;  487.1373[M-H-C_6_H_10_O_5_-C_6_H_10_O_5_]-;  405.1184[M-H-C_20_H_22_O_9_]^-^;  243.0653[M-H-C_20_H_22_O_9_-C_6_H_10_O_5_]^-^ | + | + | + | Stilbene derivatives | / |
| 73 | D8 | 8.33 | Isomer of Polygonumoside C/D | C_40_H_43_O_19_^-^ | 827.2396 | 827.2404 | 1.0 | 665.1895[M-H-C_6_H_10_O_5_]^-^;  647.1748[M-H-C_6_H_10_O_5_-H_2_O]^-^; | + | + | + | Stilbene derivatives | (Wang et al., 2017) |
| 74 | D9^a^ | 9.18 | Isomer of Multiflorumiside A | C_40_H_43_O_18_^-^ | 811.2488 | 811.2455 | -4.1 | 649.1924[M-H-C_6_H_10_O_5_]-;  405.1199[M-H-C_20_H_22_O_9_]^-^;  243.0654[M-H-C_20_H_22_O_9_-C_6_H_10_O_5_]^-^ | + | - | + | Stilbene derivatives | / |
| 75 | D10 | 9.69 | 2,3,5,4’-tetrahydroxystilbene-2-O-*β*-(3-O-monogalloyl ester)-glucoside | C_27_H_25_O_13_^-^ | 557.1299 | 557.1301 | 0.4 | 405.1192[M-H-C_7_H_4_O_4_]^-^;  313.0571[M-H-C_14_H_12_O_4_]^-^  243.0665[M-H-C_7_H_4_O_4_-C_6_H_10_O_5_]^-^;  169.0151[M-H-C_14_H_12_O_4_-C_6_H_8_O_4_]^-^ | - | + | + | Stilbene derivatives | (Xu et al., 2006, Qiu et al., 2013) |
| 76 | D11^a^ | 9.84 | Isomer of Multiflorumiside A | C_40_H_43_O_18_^-^ | 811.2477 | 811.2455 | -2.7 | 405.1222[M-H-C_20_H_22_O_9_]^-^;  243.0662[M-H-C_20_H_22_O_9_-C_6_H_10_O_5_]^-^ | + | + | + | Stilbene derivatives | / |
| 77 | D12^a^ | 9.98 | Cis-2,3,5,4’-tetrahydroxystilbene-2-O-D-glucoside | C_20_H_21_O_9_^-^ | 405.1191 | 405.1191 | 0.0 | 243.0663[M-H-C_6_H_10_O_5_]^-^ | + | + | + | Stilbene derivatives | / |
| 78 | D13 | 10.37 | Tetrahydroxystilbene-di-O-glycopyranoside | C_26_H_31_O_14_^-^ | 567.1715 | 567.1719 | 0.7 | 449.0726[M-H-C_4_H_6_O_4_]^-^;  287.0197[M-H-C_4_H_6._O_4_-C_6_H_10_O_5_]^-^;  243.0661[M-H-C_4_H_6_O_4_-C_6_H_10_O_5_-C_2_H_4_O]^-^ | + | - | - | Stilbene derivatives | (Qiu et al., 2013) |
| 79 | D14^a^ | 10.40 | Isomer of Polygonibene A | C_40_H_41_O_18_^-^ | 809.2298 | 809.2298 | 0.0 | 647.1777[M-H-C_6_H_10_O_5_]^-^；  485.1245[M-H-C_6_H_10_O_5_-C_6_H_10_O_5_]^-^ | + | + | + | Stilbene derivatives | / |
| 80 | D15 | 10.86 | Isomer of Tetrahydroxystilbene-di-O-glycopyranoside | C_26_H_31_O_14_^-^ | 567.1715 | 567.1719 | 0.7 | 449.0730[M-H-C_4_H_6_O_4_]^-^;  405.1194[M-H-C_6_H_10_O_5_]^-^;  287.0197[M-H-C_4_H_6._O_4_-C_6_H_10_O_5_]^-^;  243.0664[M-H-C_4_H_6_O_4_-C_6_H_10_O_5_-C_2_H_4_O]^-^ | + | + | + | Stilbene derivatives | (Qiu et al., 2013) |
| 81 | D16^a^ | 11.05 | Polydatin/resveratrol-hexose/piceid | C_20_H_22_O_8_^-^ | 389.1242 | 389.1231 | -2.8 | 227.0716[M-H-C_6_H_10_O_5_]^-^; | - | - | + | Stilbene derivatives | / |
| 82 | D17 | 11.17 | Isomer of 2,3,5,4’-tetrahydroxystilbene-2-O-*β*-(3-O-monogalloyl )-glucoside | C_27_H_25_O_13_^-^ | 557.1299 | 557.1301 | 0.4 | 405.1190[M-H-C_7_H_4_O_4_]^-^;  313.0565[M-H-C_14_H_12_O_4_]^-^  243.0663[M-H-C_7_H_4_O_4_-C_6_H_10_O_5_]^-^;  169.0142[M-H-C_14_H_12_O_4_-C_6_H_8_O_4_]^-^ | - | + | + | Stilbene derivatives | (Qiu et al., 2013) |
| 83 | D18^a^ | 11.51 | Trans-2,3,5,4’-tetrahydroxystilbene-2-O-D-glucoside | C_20_H_21_O_9_^-^ | 405.1191 | 405.1192 | 0.2 | 243.0668[M-H-C_6_H_10_O_5_]^-^ | + | + | + | Stilbene derivatives | / |
| 84 | D19^a^ | 11.52 | Isomer of Multiflorumiside A | C_40_H_43_O_18_^-^ | 811.2454 | 811.2455 | 0.1 | 649.1901[M-H-C_6_H_10_O_5_]-;  487.1373[M-H-C_6_H_10_O_5_-C_6_H_10_O_5_]-;  405.1190[M-H-C_20_H_22_O_9_]^-^;  243.0664[M-H-C_20_H_22_O_9_-C_6_H_10_O_5_]^-^ | - | + | - | Stilbene derivatives | / |
| 85 | D20 | 12.23 | Isomer of 2,3,5,4’-tetrahydroxystilbene-2-O-*β*-(3-O-monogalloyl ester)-glucoside | C_27_H_25_O_13_^-^ | 557.1299 | 557.1301 | 0.4 | 405.1188[M-H-C_7_H_4_O_4_]^-^;  243.0667[M-H-C_7_H_4_O_4_-C_6_H_10_O_5_]^-^;  313.0570M-H-C_14_H_12_O_4_]^-^;  169.0143[M-H-C_14_H_12_O_4_-C_6_H_8_O_4_]^-^ | - | + | + | Stilbene derivatives | (Xu et al., 2006, Qiu et al., 2013) |
| 86 | D21 | 12.51 | Tetrahydroxystilbene-O-(acetyl)-hexose | C_22_H_3_O_10_^-^ | 447.1297 | 447.1297 | 0.0 | 405.1188[M-H-C_2_H_2_O]^-^  243.0661[M-H-C_2_H_2_O-C_6_H_10_O_5_]^-^； | - | + | + | Stilbene derivatives | (Wang et al., 2017) |
| 87 | D22^a^ | 13.06 | Polygonibene A | C_40_H_41_O_18_^-^ | 809.2298 | 809.2298 | 0.0 | 647.1777[M-H-C_6_H_10_O_5_]^-^；  485.1245[M-H-C_6_H_10_O_5_-C_6_H_10_O_5_]^-^ | + | + | + | Stilbene derivatives | / |
| 88 | D23 | 13.63 | Isomer of Tetrahydroxystilbene-O-(acetyl)-hexose | C_22_H_3_O_10_^-^ | 447.1292 | 447.1297 | 1.1 | 405.1188[M-H-C_2_H_2_O]^-^  243.0664[M-H-C_2_H_2_O-C_6_H_10_O_5_]^-^； | + | + | - | Stilbene derivatives | (Wang et al., 2017) |
| 89 | D24 | 13.74 | 2,3,5,4'-tetrahydroxystilbene-2-O-(Caffeoyl)-glucopyranosside | C_26_H_31_O_14_^-^ | 567.1506 | 567.1508 | 0.4 | 405.1206[M-H-C_6_H_10_O_5_]^-^；  243.0660[M-H-C_6_H_10_O_5_-C_6_H_10_O_5_]^-^；  161.0243[M-H-C_15_H_24_O_10_-H_2_O]^-^ | - | + | + | Stilbene derivatives | (Qiu et al., 2013) |
| 90 | D25^a^ | 13.58 | Polygonibene B | C_40_H_41_O_18_^-^ | 809.2288 | 809.2298 | 1.2 | 647.1813[M-H-C_6_H_10_O_5_]^-^；  485.1237[M-H-C_6_H_10_O_5_-C_6_H_10_O_5_]^-^ | + | + | + | Stilbene derivatives | / |
| 91 | D26 | 13.88 | Resveratrol | C_14_H_11_O_3_^-^ | 227.0714 | 227.0714 | 0.0 | 188.0479[M-H-CO-CH_3_]^-^ | - | + | - | Stilbene derivatives | (Zhang 2016) |
| 92 | D27^a^ | 14.45 | Isomer of Polygonibene A | C_40_H_41_O_18_^-^ | 809.2288 | 809.2298 | 1.2 | 647.1777[M-H-C_6_H_10_O_5_]^-^；  485.1245[M-H-C_6_H_10_O_5_-C_6_H_10_O_5_]^-^ | + | + | + | Stilbene derivatives | / |
| 93 | D28 | 14.62 | Polygonumoside A | C_27_H_23_O_13_^-^ | 555.1136 | 555.1144 | 1.4 | 393.0615[M-H-C_6_H_10_O_5_]^-^；  243.0669[M-H-C_7_H_2_O_4_]^-^ | + | + | - | Stilbene derivatives | (Wang et al., 2021) |
| 94 | D29 | 14.91 | Isomer of Tetrahydroxystilbene-O-(malonyl)-hexose | C_23_H_23_O_12_^-^ | 491.1190 | 491.1195 | 1.0 | 405.1190[M-H-C_3_H_2_O_3_]^-^;  243.0670[M-H-C_3_H_2_O_3_-C_6_H_10_O_5_]^-^; | - | + | + | Stilbene derivatives | (Wang et al., 2017) |
| 95 | D30 | 15.39 | Isomer of Polygonumoside A | C_27_H_23_O_13_^-^ | 555.1136 | 555.1144 | 1.4 | 393.0615[M-H-C_6_H_10_O_5_]^-^；  243.0669[M-H-C_7_H_2_O_4_]^-^ | + | + | - | Stilbene derivatives | (Wang et al., 2021) |
| 96 | D31 | 15.51 | Tetrahydroxystilbene-O-(coumaroyl)-hexose | C_29_H_27_O_11_^-^ | 551.1555 | 551.1559 | 0.7 | 405.1190[M-H-C_9_H_6_O_2_]^-^;  243.0670[M-H-C_9_H_6_O_2_-C_6_H_10_O_5_]^-^; | + | + | + | Stilbene derivatives | (Wang et al., 2014, Wang et al., 2017) |
| 97 | D32^a^ | 15.55 | Tetrahydroxystilbene-O-(feruloyl)-hexose | C_20_H_29_O_12_^-^ | 581.1661 | 581.1664 | 0.5 | 405.1187[M-H-C_10_H_8_O_3_]^-^;  387.1090[M-H-C_10_H_8_O_3_-H_2_O]^-^;  337.0918[M-H-C_14_H_12_O_4_]^-^;  243.0661[M-H-C_10_H_8_O_3_-C_6_H_10_O_5_]^-^;  175.0404[M-H-C_10_H_22_O_9_]^-^ | - | + | + | Stilbene derivatives | / |
| 98 | D33 | 15.62 | Isomer of Tetrahydroxystilbene-O-(feruloyl)-hexose | C_20_H_29_O_12_^-^ | 581.1657 | 581.1664 | 1.2 | 405.1207[M-H-C_10_H_8_O_3_]^-^;  337.0927[M-H-C_10_H_22_O_9_]^-^;  243.0660[M-H-C_10_H_8_O_3_-C_6_H_10_O_5_]^-^;  175.0409[M-H-C_10_H_22_O_9_]^-^ | + | - | - | Stilbene derivatives | (Wang et al., 2017) |
| 99 | A1 | 9.87 | Emodin acid-O-hexose | C_22_H_23_O_12_^-^ | 479.1189 | 479.1195 | 1.3 | 299.0552[M-H-C_6_H_12_O_6_]^-^;  255.0652[M-H-C_6_H_12_O_6_-CO_2_]^-^; | + | + | + | Anthraquinones | (Qiu et al., 2013) |
| 100 | A2 | 9.84 | Isomer of emodin-emodin dianthrone-di-glucopyranoside | C_42_H_41_O_18_^-^ | 833.2298 | 833.2298 | 0.0 | 671.1759[M-H-C_6_H_12_O_6_]^-^;  509.1180[M-H-C_6_H_12_O_6_-C_6_H_12_O_6_]^-^ | + | + | + | Anthraquinones | (Yang et al., 2019) |
| 101 | A3 | 13.11 | Cassialoin | C_21_H_21_O_9_^-^ | 417.1195 | 417.1191 | 1.0 | 255.0656[M-H-C_6_H_10_O_5_]^-^ | - | + | + | Anthraquinones | (Luo et al., 2017) |
| 102 | A4 | 14.16 | Isomer of Cassialoin | C_21_H_21_O_9_^-^ | 417.1191 | 417.1191 | 0.0 | 255.0664[M-H-C_6_H_10_O_5_]^-^ | - | + | + | Anthraquinones | (Luo et al., 2017) |
| 103 | A5 | 15.14 | Citreorosein-O-glucopyranoside | C_21_H_19_O_11_^-^ | 447.0932 | 447.0933 | 0.2 | 493.0973[M-H+CH_2_O]^-^  327.0526[M-H-C_4_H_8_O_4_]^-^;  285.0430[M-H-C_4_H_8_O_4_-C_2_H_2_O]^-^ | - | + | - | Anthraquinones | (Wang et al., 2015) |
| 104 | A6 | 15.68 | Emodin-8-O-*β*-hexose-sulphate | C_21_H_19_O_13_^-^ | 511.0550 | 511.0552 | 0.4 | 431.0982[M-H-SO_3_]^-^;  349.0720[M-H-C_6_H_10_O_5_]^-^;  269.0454[M-H-SO_3_-C_6_H_10_O_5_]^-^ | + | + | + | Anthraquinones | (Wang et al., 2017) |
| 105 | A7 | 17.56 | Emodin-1-O-*β*-glucoside | C_21_H_19_O_10_^-^ | 431.0982 | 431.0984 | 0.5 | 269.0454[M-H-C_6_H_10_O_5_]^-^;  240.0426[M-H-C_6_H_10_O_5_-CHO]^-^ | + | + | + | Anthraquinones | / |
| 106 | A8 | 19.83 | Polygonumnolide B1 | C_43_H_43_O_18_^-^ | 847.2455 | 847.2455 | 0.0 | 685.1929[M-H-C_6_H_10_O_5_]^-^;  523.1458[M-H-C_6_H_10_O_5_-C_6_H_10_O_5_]^-^ | - | + | + | Anthraquinones | (Yang et al., 2019) |
| 107 | A9 | 20.02 | Fallacinol/questinol | C_16_H_11_O_6_^-^ | 299.0561 | 299.0561 | 0.0 | 284.0335[M-H-CH_3_]^-^;  256.0378[M-H-CH_3_-CO]^-^ | + | + | + | Anthraquinones | (Qiu et al., 2013) |
| 108 | A10 | 20.68 | Acetyl-emodin-glucopyranoside | C_23_H_21_O_11_^-^ | 473.1088 | 473.1089 | 0.2 | 311.0554[M-H-C_6_H_10_O_5_]^-^;  269.0462[M-H-C_6_H_10_O_5_-C_2_H_2_O]^-^ | - | + | + | Anthraquinones | (Wang et al., 2021) |
| 109 | A11 | 21.73 | Isomer of emodin-emodin dianthrone-di-glucopyranoside | C_42_H_41_O_18_^-^ | 833.2298 | 833.2298 | 0.0 | 671.1759[M-H-C_6_H_12_O_6_]^-^;  509.1180[M-H-C_6_H_12_O_6_-C_6_H_12_O_6_]^-^ | - | + | + | Anthraquinones | (Yang et al., 2019) |
| 110 | A12 | 22.02 | Emodin-8-O-*β*-glucoside | C_21_H_19_O_10_^-^ | 431.0982 | 431.0984 | 0.5 | 311.0560[M-H-C_4_H_8_O_4_]^-^;  269.0456[M-H-C_6_H_10_O_5_]^-^; | + | + | + | Anthraquinones | / |
| 111 | A13 | 22.76 | Endocrocin | C_16_H_9_O_7_^-^ | 313.0355 | 313.0354 | 0.3 | 299.0214[M-H-CH_2_]^-^;  269.0456[M-H-CO_2_]^-^; | - | + | - | Anthraquinones | (Huang et al., 2018) |
| 112 | A14 | 23.070 | Emodin-O-(malonyl)-glucopyranoside | C_24_H_21_O_13_^-^ | 517.0988 | 517.0988 | 0.0 | 473.1095[M-H-CO_2_]^-^;  431.0989[[M-H-C_3_H_2_O_2_]^-^  269.0454[M-H-C_8_H_12_O_6_]^-^ | - | + | + | Anthraquinones | (Wang et al., 2017) |
| 113 | A15 | 24.07 | Isomer of Acetyl-emodin-glucopyranoside | C_23_H_21_O_11_^-^ | 473.1088 | 473.1089 | 0.2 | 311.0546[M-H-C_6_H_10_O_5_]^-^;  269.0454M-H-C_6_H_10_O_5_-C_2_H_2_O]^-^ | - | + | + | Anthraquinones | (Wang et al., 2021) |
| 114 | A16 | 24.62 | Isomer of Polygonumnolide B1 | C_43_H_43_O_18_^-^ | 847.2455 | 847.2455 | 0.0 | 685.1929[M-H-C_6_H_10_O_5_]^-^;  523.1458[M-H-C_6_H_10_O_5_-C_6_H_10_O_5_]^-^ | - | + | + | Anthraquinones | (Yang et al., 2019) |
| 115 | A17^a^ | 24.64 | Physicion-8-O-glucopyranoside | C_15_H_9_O_16_^-^ | 445.1144 | 445.1140 | 0.9 | 283.0614[M-H-C_6_H_10_O_5_]^-^;  241.1056[M-H-C_6_H_10_O_5_-CH_3_]^-^; | - | + | + | Anthraquinones | / |
| 116 | A18 | 24.78 | Emodin-emodin dianthrone-di-glucopyranoside | C_42_H_41_O_18_^-^ | 833.2298 | 833.2298 | 0.0 | 671.1759[M-H-C_6_H_12_O_6_]^-^;  509.1180[M-H-C_6_H_12_O_6_-C_6_H_12_O_6_]^-^ | - | + | + | Anthraquinones | (Yang et al., 2019) |
| 117 | A19 | 24.84 | Citreorosein | C_15_H_9_O_16_^-^ | 285.0404 | 285.0405 | 0.4 | 257.0453[M-H-CO]^-^;  241.0503[M-H-CO_2_]^-^ | + | + | + | Anthraquinones | (Huang et al., 2018) |
| 118 | A20 | 25.19 | Isomer of Emodin-emodin dianthrone-di-glucopyranoside | C_42_H_41_O_18_^-^ | 833.2298 | 833.2298 | 0.0 | 671.1759[M-H-C_6_H_12_O_6_]^-^;  509.1180[M-H-C_6_H_12_O_6_-C_6_H_12_O_6_]^-^ | - | + | + | Anthraquinones | (Yang et al., 2019) |
| 119 | A21 | 25.74 | Isomer of emodin-8-methyl ether/Questin | C_16_H_11_O_5_^-^ | 283.0613 | 283.0612 | 0.4 | 268.0372[M-H-CH_3_]^-^;  240.0430[M-H-C_3_H_2_O]^-^ | - | - | + | Anthraquinones | (Wang et al., 2021) |
| 120 | A22 | 25.86 | Isomer of Polygonumnolide B1 | C_43_H_43_O_18_^-^ | 847.2455 | 847.2455 | 0.0 | 685.1929[M-H-C_6_H_10_O_5_]^-^;  523.1458[M-H-C_6_H_10_O_5_-C_6_H_10_O_5_]^-^ | - | + | + | Anthraquinones | (Yang et al., 2019) |
| 121 | A23 | 25.93 | Isomer of acetyl-emodin | C_17_H_11_O_6_^-^ | 311.0562 | 311.0561 | 0.3 | 283.0249[M-H-CO]^-^; | - | + | - | Anthraquinones | (Wang et al., 2021) |
| 122 | A24 | 26.09 | Physcion-physcion bianthrone glucopyranoside | C_38_H_35_O_13_^-^ | 699.2083 | 699.2083 | 0.0 | 537.1555[M-H-C_6_H_10_O_5_]^-^;  431.0952[M-H-C_16_H_12_O_4_]^-^;  269.0773[M-H-C_16_H_12_O_4_-C_6_H_10_O_5_]^-^ | - | + | + | Anthraquinones | (Yang et al., 2019) |
| 123 | A25 | 26.43 | Isomer of Polygonumnolide B1 | C_43_H_43_O_18_^-^ | 847.2455 | 847.2455 | 0.0 | 685.1929[M-H-C_6_H_10_O_5_]^-^;  523.1458[M-H-C_6_H_10_O_5_-C_6_H_10_O_5_]^-^ | - | + | + | Anthraquinones | (Yang et al., 2019) |
| 124 | A26 | 26.49 | Emodin-emodin dianthrone-glucopyranoside | C_36_H_31_O_13_^-^ | 671.1770 | 671.1770 | 0.0 | 416.1111[M-H-C_15_H_11_O_4_]^-^;  509.1244[M-H-C_6_H_12_O_6_]^-^ | - | + | + | Anthraquinones | (Yang et al., 2019) |
| 125 | A27 | 26.59 | Physcion physcion-glucopyranoside | C_38_H_35_O_13_^-^ | 699.2083 | 699.2083 | 0.0 | 537.1555[M-H-C_6_H_10_O_5_]^-^;  431.0952[M-H-C_16_H_12_O_4_]^-^;  269.0773[M-H-C_16_H_12_O_4_-C_6_H_10_O_5_]^-^ | - | + | + | Anthraquinones | (Yang et al., 2019) |
| 126 | A28 | 26.74 | Isomer of Emodin-emodin dianthrone-glucopyranoside | C_36_H_31_O_13_^-^ | 671.1770 | 671.1770 | 0.0 | 416.1111[M-H-C_15_H_11_O_4_]^-^;  509.1244[M-H-C_6_H_12_O_6_]^-^ | - | + | + | Anthraquinones | (Yang et al., 2019) |
| 127 | A29 | 27.28 | emodin-8-methyl ether/Questin | C_16_H_11_O_5_^-^ | 283.0616 | 283.0612 | 1.4 | 240.0436[M-H-C_3_H_2_O]^-^ | - | + | + | Anthraquinones | (Wang et al., 2021) |
| 128 | A30 | 28.40 | Acetyl-emodin | C_17_H_11_O_6_^-^ | 311.0562 | 311.0561 | 0.3 | 283.0249[M-H-CO]^-^; | - | + | - | Anthraquinones | (Wang et al., 2021) |
| 129 | A31 | 27.48 | Emodin-physcion dianthrone-glucopyranoside | C_37_H_33_O_13_^-^ | 685.2002 | 685.1927 | 10.9 | 254.0587[M-H-C_21_H_21_O_9_]^-^ | - | + | + | Anthraquinones | (Yang et al., 2019) |
| 130 | A32 | 27.81 | Isomer of Emodin-physcion dianthrone-glucopyranoside | C_37_H_33_O_13_^-^ | 685.1916 | 685.1927 | 1.6 | 254.0583[M-H-C_22_H_23_O_9_]^-^ | - | + | + | Anthraquinones | (Yang et al., 2019) |
| 131 | A33 | 27.80 | Isomer of Emodin-emodin dianthrone-glucopyranoside | C_36_H_31_O_13_^-^ | 671.1770 | 671.1770 | 0.0 | 416.1111[M-H-C_15_H_11_O_4_]^-^;  509.1244[M-H-C_6_H_12_O_6_]^-^ | - | + | + | Anthraquinones | (Yang et al., 2019) |
| 132 | A34 | 28.14 | Isomer of Emodin-emodin dianthrone-glucopyranoside | C_36_H_31_O_13_^-^ | 671.1770 | 671.1770 | 0.0 | 416.1111[M-H-C_15_H_11_O_4_]^-^;  509.1244[M-H-C_6_H_12_O_6_]^-^ | - | + | + | Anthraquinones | (Yang et al., 2019) |
| 133 | A35 | 28.31 | Isomer of Emodin-physcion dianthrone-glucopyranoside | C_37_H_33_O_13_^-^ | 685.1969 | 685.1927 | 6.1 | 254.0583[M-H-C_22_H_23_O_9_]^-^ | - | + | + | Anthraquinones | (Yang et al., 2019) |
| 134 | A36 | 28.64 | Isomer of Emodin-physcion dianthrone-glucopyranoside | C_37_H_33_O_13_^-^ | 685.1896 | 685.1927 | 4.5 | 254.0586[M-H-C_22_H_23_O_9_]^-^ | - | + | + | Anthraquinones | (Yang et al., 2019) |
| 135 | A37 | 28.88 | Isomer of Emodin-physcion dianthrone-glucopyranoside | C_37_H_33_O_13_^-^ | 685.1962 | 685.1927 | 5.1 | 416.1116[M-H-C_16_H_13_O_4_]^-^ | - | + | + | Anthraquinones | (Yang et al., 2019) |
| 136 | A38 | 29.05 | Isomer of Emodin-physcion dianthrone-glucopyranoside | C_37_H_33_O_13_^-^ | 685.1929 | 685.1927 | 0.3 | 416.1123[M-H-C_16_H_13_O_4_]^-^ | - | + | + | Anthraquinones | (Yang et al., 2019) |
| 137 | A39 | 29.30 | Isomer of Emodin-physcion dianthrone-glucopyranoside | C_37_H_33_O_13_^-^ | 685.1927 | 685.1927 | 0.0 | 416.1114[M-H-C_16_H_13_O_4_]^-^ | - | + | + | Anthraquinones | (Yang et al., 2019) |
| 138 | A40 | 29.63 | Isomer of Emodin-physcion dianthrone-glucopyranoside | C_37_H_33_O_13_^-^ | 685.1927 | 685.1927 | 0.0 | 416.1114[M-H-C_16_H_13_O_4_]^-^ | - | + | + | Anthraquinones | (Yang et al., 2019) |
| 139 | A41^a^ | 29.89 | emodin | C_15_H_9_O_5_^-^ | 269.0455 | 269.0456 | 0.4 | 241.0506[M-H-CO]^-^;  225.0560[M-H-CO-CO]^-^; | + | + | + | Anthraquinones | / |
| 140 | A42 | 30.21 | Isomer of physcion physcion glucopyranoside | C_38_H_35_O_13_^-^ | 699.2090 | 699.2083 | 1.0 | 537.1555[M-H-C_6_H_10_O_5_]^-^;  431.0952[M-H-C_16_H_12_O_4_]^-^;  269.0773[M-H-C_16_H_12_O_4_-C_6_H_10_O_5_]^-^ | - | + | + | Anthraquinones | (Yang et al., 2019) |
| 141 | A43 | 30.26 | Emodin-emodin dianthrone | C_30_H_21_O_8_^-^ | 509.1241 | 509.1242 | 0.2 | 254.0583[M-H-C_15_H_11_O_4_]^-^;  467.1948[M-H-CO_2_]- | - | + | + | Anthraquinones | (Yang et al., 2019) |
| 142 | A44 | 30.71 | Isomer of physcion-physcion glucopyranoside | C_38_H_35_O_13_^-^ | 699.2093 | 699.2083 | 1.4 | 537.1555[M-H-C_6_H_10_O_5_]^-^;  431.0952[M-H-C_16_H_12_O_4_]^-^;  269.0773[M-H-C_16_H_12_O_4_-C_6_H_10_O_5_]^-^ | - | + | + | Anthraquinones | (Yang et al., 2019) |
| 143 | A45 | 31.00 | Isomer of emodin-emodin dianthrone | C_30_H_21_O_8_^-^ | 509.1241 | 509.1242 | 0.2 | 254.0583[M-H-C_15_H_11_O_4_]^-^;  467.1948[M-H-CO_2_]- | - | + | + | Anthraquinones | (Yang et al., 2019) |
| 144 | A46 | 32.95 | Isomer of citreorosein  (Fesetin) | C_15_H_9_O_16_^-^ | 285.0406 | 285.0405 | 0.4 | 257.0457[M-H-CO]^-^;  241.0500[M-H-CO_2_]^-^ | - | + | + | Anthraquinones | (Huang et al., 2018) |
| 145 | A47^a^ | 34.06 | Cis-emodin-physcion bianthron | C_31_H_23_O_8_^-^ | 523.1399 | 523.1390 | 1.7 | 254.0583[M-H-C_16_H_13_O_4_]^-^; | - | + | + | Anthraquinones | / |
| 146 | A48^a^ | 34.39 | Isomer of Cis-emodin-phscion bianthron | C_31_H_23_O_8_^-^ | 523.1399 | 523.1404 | 1.0 | 254.0583[M-H-C_16_H_13_O_4_]^-^; | - | + | + | Anthraquinones | / |
| 147 | A49 | 38.36 | Physcion-physcion bianthrone | C_32_H_25_O_8_^-^ | 537.1577 | 537.1555 | 4.1 | 269.0773[M-H-C_16_H_12_O_4_]^-^ | - | + | + | Anthraquinones | (Yang et al., 2019) |
| 148 | A50 | 38.70 | Isomer of Physcion-physcion bianthrone | C_32_H_25_O_8_^-^ | 537.1540 | 537.1555 | 2.8 | 269.0773[M-H-C_16_H_12_O_4_]^-^ | - | + | + | Anthraquinones | (Yang et al., 2019) |
| 149 | B1^a^ | 8.19 | N,N-dimethyl-tryptophan methylester | C_14_H_17_N_2_O_2_^-^ | 245.1296 | 245.1296 | 0.0 | 142.0663[M-H-C_2_H_3_O_2-_C_2_H_6_N]^-^;  186.0560[M-H-C_2_H_3_O_2_]^-^; | - | + | - | Alkaloids | / |
| 150 | B2^a^ | 10.75 | P-hydroxybenzal-dehyde | C_7_H_5_O_2_^-^ | 121.0295 | 121.0295 | 0.0 |  | + | + | + | Alkaloids | / |
| 151 | B3^a^ | 15.51 | N-trans-Feruloyltyramine | C_18_H_18_NO_4_^-^ | 312.1241 | 312.1242 | -0.3 | 297.1007[M-H-CH_3_]^-^;  178.0510[M-H-C_8_H_6_O_2_]^-^;  148.0530[M-H-C_9_H_10_NO_2_]^-^ | - | + | - | Alkaloids | / |
| 152 | B4^a^ | 15.72 | N-trans-Feruloyl-3-methyldopamine | C_19_H_20_NO_5_^-^ | 342.1345 | 342.1347 | -0.6 | 327.1112[M-H-CH_3_]^-^; 178.0508[M-H-CH_3_-C_10_H_13_O_2_]^-^;  148.0528 [M-H-C_10_H_12_NO_3_]^-^ | - | + | - | Alkaloids | / |

Note: H_2_O: aqueous extract of PMR; 70% EtOH: 70% ethanol extract of PMR; 95% EtOH: 95% ethanol extract of PMR.

a means components compared with standards. b means components means first reported in PMR. n means potential new compound.

1. **Supplementary Figures**


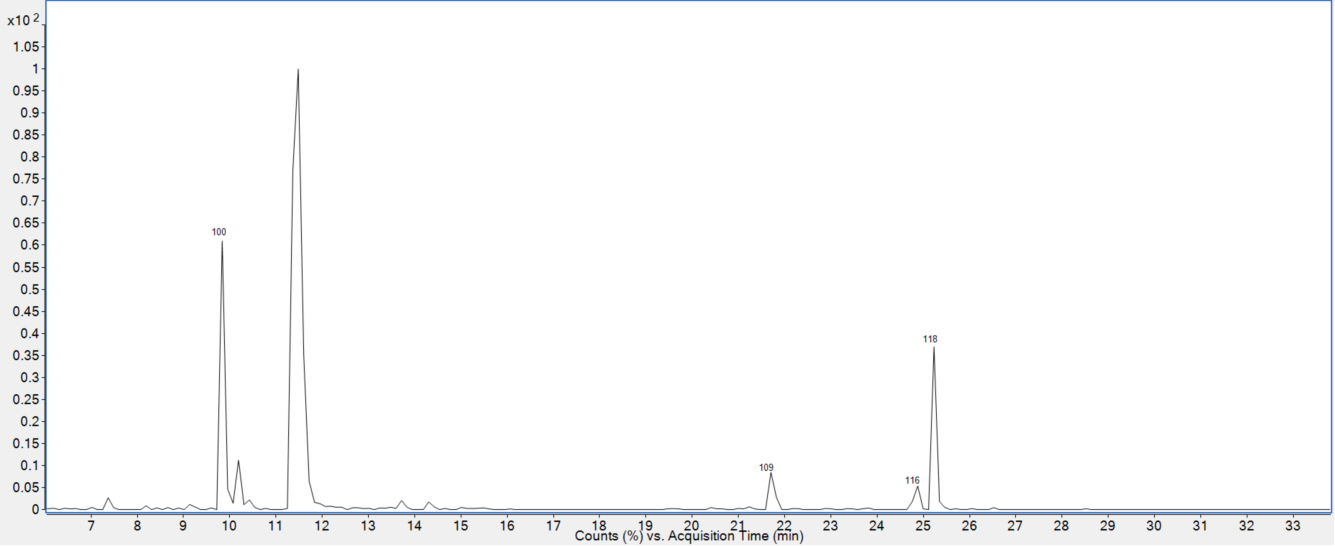


A

B

C

D

E

F

G

H

I

J

K


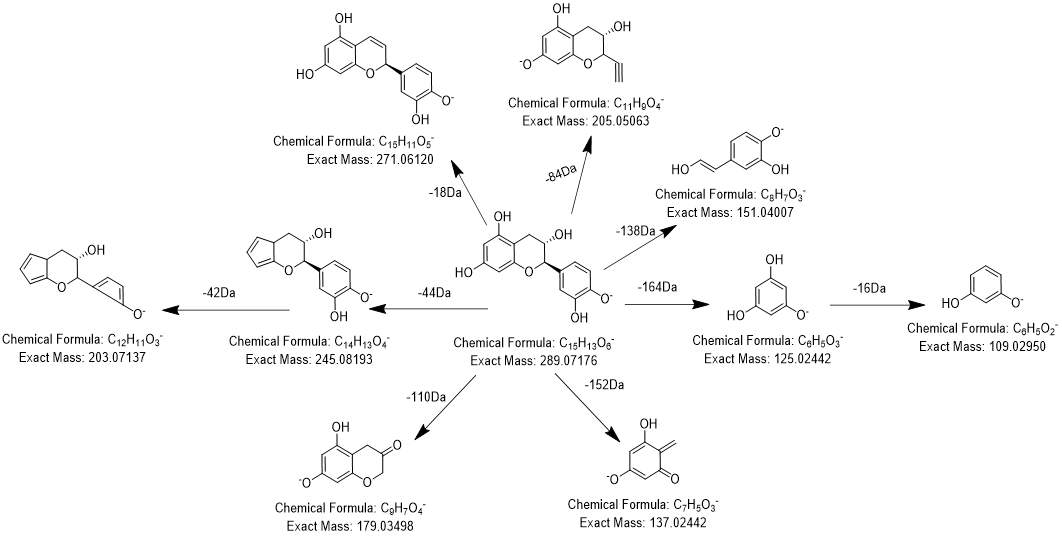
**Supplementary Figure 1** the extraction chromatograms of *m/z* 833.2298(A), *m/z* 699.2083 (B), *m/z* 685.1927 (C), *m/z* 671.1770 (D), *m/z* 509.1242 (E), *m/z* 523.1390 (F) and *m/z* 537.1555 (G); BPC of standard alkaloids (H); BPC of standard anthraquinones (I); BPC of standard stilbene derivatives (J); BPC of other standards (K).

**Supplementary Figure 2** The proposed fragmentation pathways of Catechin

**
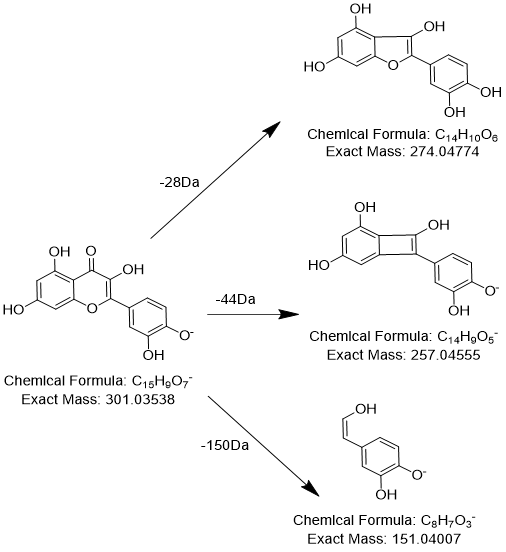
**

**Supplementary Figure 3** The proposed fragment path way of Quercetin.


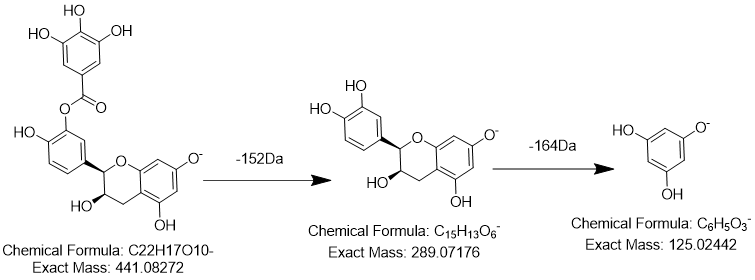


**Supplementary Figure 3** The proposed fragmentation pathways of **FL14** (catechin galloyl easter)

**Supplementary Figure 5** The proposed fragmentation pathways of **FL16 (**Polygonflavanol A)

**Supplementary Figure 6** The proposed fragmentation pathways of **FL1 (**gallocatechin)

**Supplementary Figure 7** The proposed fragmentation pathways of **FL11 (**Dihydrokaempferol-3-glucoside)

**Supplementary Figure 8** The proposed fragmentation pathways of **FL19 (**naringenin-7-O-β-glucuronide)

**Supplementary Figure 9** The proposed fragmentation pathways of **FL2 (**procyanidin B)

**Supplementary Figure 10** The proposed fragmentation pathways of **FL9 (**di-O-galloyl-procyanidin B2)

**Supplementary Figure 11** The proposed fragmentation pathways of **FL3 (**procyanidin C1)


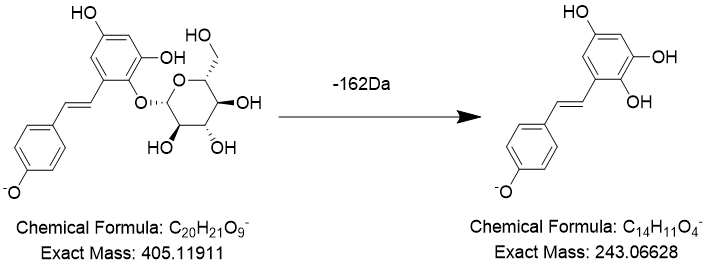


**Supplementary Figure 12** The proposed fragmentation pathways of trans-2,3,5,4’-tetrahydroxystilbene-2-O-D-glucoside


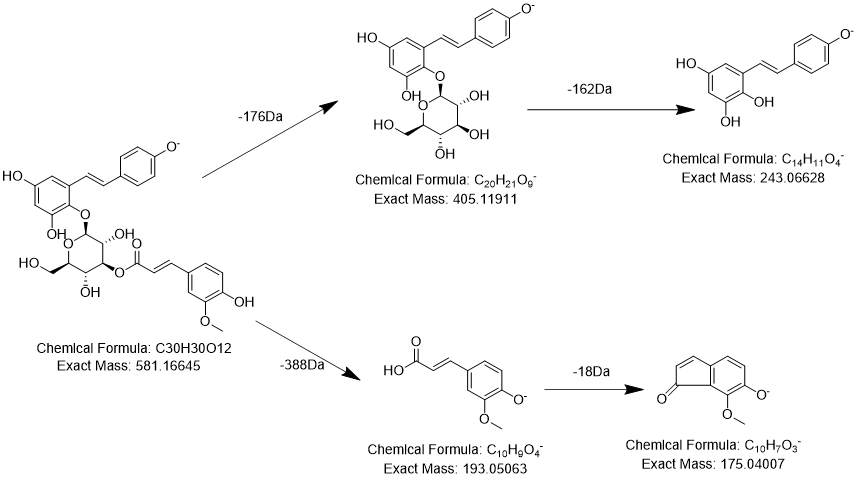


**Supplementary Figure 13** The proposed fragmentation pathways of 2,3,5,4'-tetrahydroxystilbene-2-O-(2''-Feruloy)-*β*-D-glucopyranoside

**Supplementary Figure 14** The proposed fragmentation pathways of Multiflorumiside A

**Supplementary Figure 15** The proposed fragmentation pathways of Polygonibene A

**Supplementary Figure 16** The proposed fragmentation pathways of Polydatin

**Supplementary Figure 17** The proposed fragmentation pathways of **D3 (**Polygonumoside C/D)

**Supplementary Figure 18** The proposed fragmentation pathways of **D10** (2,3,5,4’-tetrahydroxystilbene-2-O-β-(3-O-monogalloyl ester)-glucoside)

·

**Supplementary Figure 19** The proposed fragmentation pathways of **D21 (**Tetrahydroxystilbene-O-(acetyl)-hexose)

**Supplementary Figure 20** The proposed fragmentation pathways of **D24** (2,3,5,4'-tetrahydroxystilbene-2-O-(Caffeoyl)-glucopyranoside)

**Supplementary Figure 21** The proposed fragmentation pathways of **D31** (Tetrahydroxystilbene-O-(coumaroyl)-hexose)

**Supplementary Figure 22** The proposed fragmentation pathways of **D29** (Tetrahydroxystilbene-O-(malonyl)-hexose)


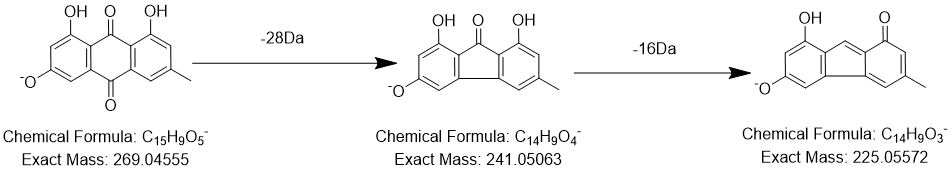


**Supplementary Figure 23** The proposed fragmentation pathways of emodin


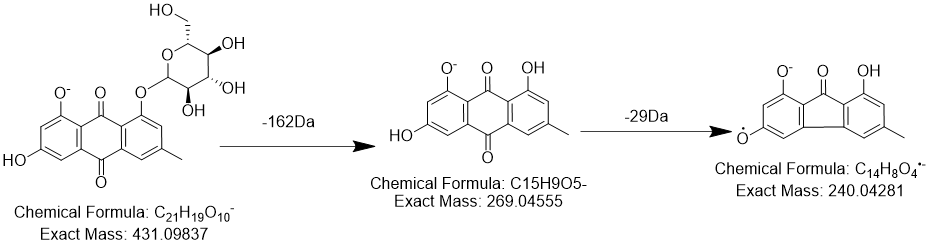


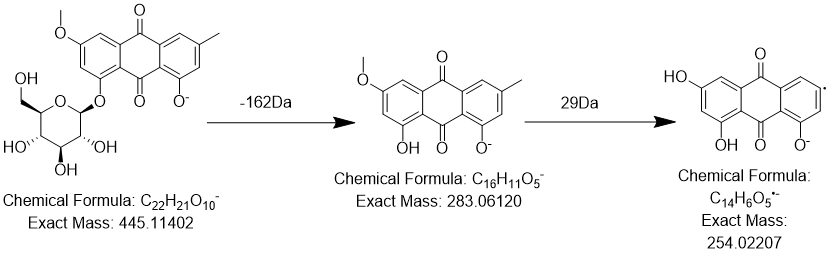
**Supplementary Figure 24** The proposed fragmentation pathways of Emodin-1-O-*β*-glucoside

**Supplementary Figure 25** The proposed fragmentation pathways of physcion-8-O-*β*-D-glucopyranoside


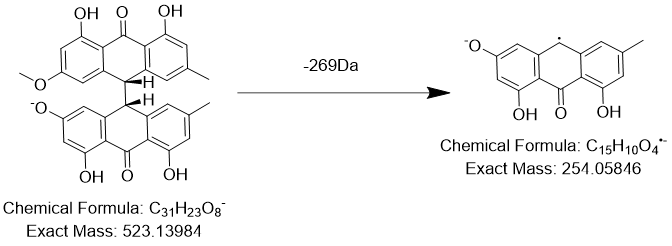


**Supplementary Figure 26**
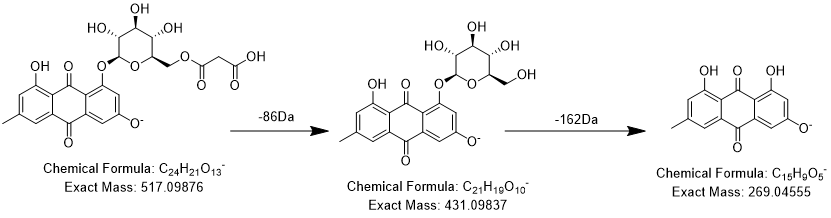
The proposed fragmentation pathways of *cis*-emodin-phscion bianthron

**Supplementary Figure 27** The proposed fragmentation pathways of **A14** (Emodin-O-(malonyl)-glucopyranoside)

**Supplementary Figure 28** The proposed fragmentation pathways of **A10** (acetyl-emodin-glucopyranoside)

**Supplementary Figure 29** The proposed fragmentation pathways of **A13** (Endocrocin)

**Supplementary Figure 30** The proposed fragmentation pathways of **A6** (emodin-8-O-*β*-D-hexose-sulphate)


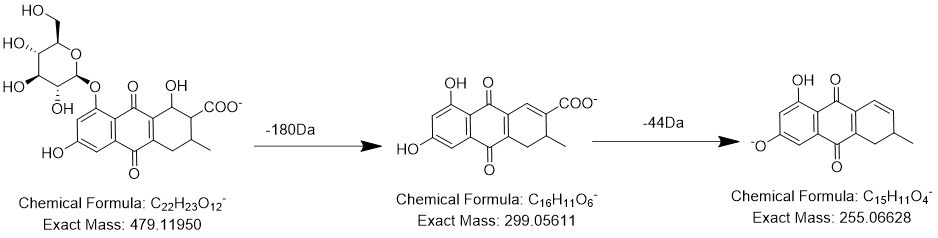


**Supplementary Figure 31** The proposed fragmentation pathways of **A1** (emodin acid-hexose)


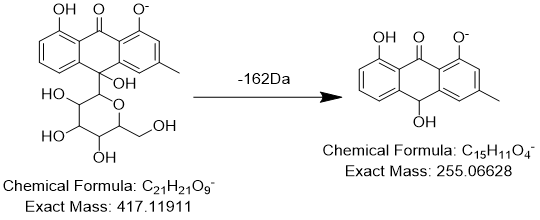


**Supplementary Figure 32** The proposed fragmentation pathways of **A3** (cassialoin)


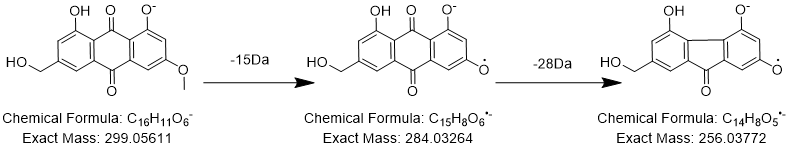


**Supplementary Figure 33** The proposed fragmentation pathways of **A9** (questinol)


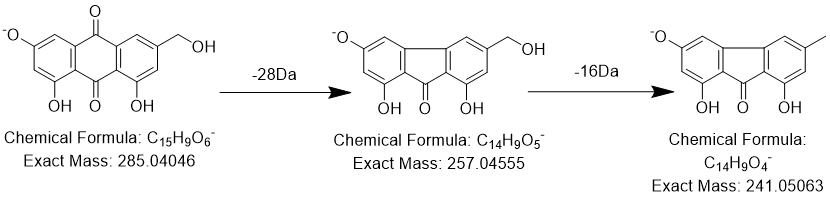


**Supplementary Figure 34** The proposed fragmentation pathways of **A19** (citreorosein)

**Supplementary Figure 35** The proposed fragmentation pathways of **A5** (citreorosein-O-glucopyranoside)

**Supplementary Figure 36** The proposed fragmentation pathways of **A21** (emodin-O-methyl ether/Questin)

**Supplementary Figure 37** The proposed fragmentation pathways of **A30** (acetyl-emodin)

**Supplementary Figure 38** The proposed fragmentation pathways of torachrysone-8-O-glucoside

**Supplementary Figure 39** The proposed fragmentation pathways of Polygonimitin E

**Supplementary Figure 40** The proposed fragmentation pathways of **N7** (torachrysone-8-O-(6’-O-acetyl)-D-glucopyranoside)


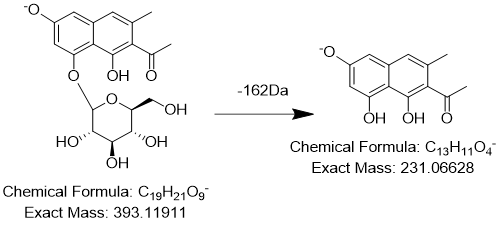


**Supplementary Figure 41** The proposed fragmentation pathways of **N1** (hydroxymusizin-O-glucopyranoside)

**Supplementary Figure 42** The proposed fragmentation pathways of **N2** (hydroxymusizin-O-glucose-O-xylose)


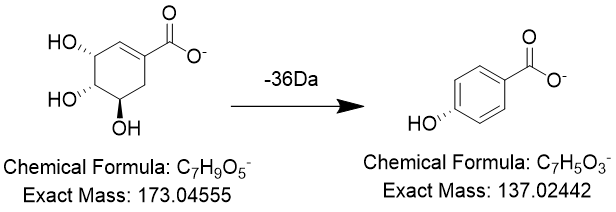


**Supplementary Figure 43** The proposed fragmentation pathways of **F1** (Shikimic acid)
